# *Akt1* Is Essential for Postnatal Mammary Gland Development, Function, and the Expression of *Btn1a1*

**DOI:** 10.1371/journal.pone.0024432

**Published:** 2011-09-07

**Authors:** Jessica LaRocca, Jodie Pietruska, Mary Hixon

**Affiliations:** Department of Pathology and Laboratory Medicine, Brown University, Providence, Rhode Island, United States of America; University of Pennsylvania School of Medicine, United States of America

## Abstract

*Akt1*, a serine-threonine protein kinase member of the *PKB/Akt* gene family, plays critical roles in the regulation of multiple cellular processes, and has previously been implicated in lactation and breast cancer development. In this study, we utilized *Akt1*+/+ and *Akt1*−/− C57/Bl6 female mice to assess the role that *Akt1* plays in normal mammary gland postnatal development and function. We examined postnatal morphology at multiple time points, and analyzed gene and protein expression changes that persist into adulthood. *Akt1* deficiency resulted in several mammary gland developmental defects, including ductal outgrowth and defective terminal end bud formation. Adult *Akt1*−/− mammary gland composition remained altered, exhibiting fewer alveolar buds coupled with increased epithelial cell apoptosis. Microarray analysis revealed that *Akt1* deficiency altered expression of genes involved in numerous biological processes in the mammary gland, including organismal development, cell death, and tissue morphology. Of particular importance, a significant decrease in expression of *Btn1a1*, a gene involved in milk lipid secretion, was observed in *Akt1*−/− mammary glands. Additionally, pseudopregnant *Akt1*−/− females failed to induce *Btn1a1* expression in response to hormonal stimulation compared to their wild-type counterparts. Retroviral-mediated shRNA knockdown of *Akt1* and *Btn1a1* in MCF-7 human breast epithelial further illustrated the importance of *Akt1* in mammary epithelial cell proliferation, as well as in the regulation of *Btn1a1* and subsequent expression of *ß-casein*, a gene that encodes for milk protein. Overall these findings provide mechanistic insight into the role of *Akt1* in mammary morphogenesis and function.

## Introduction

The mammary gland is a complex structure composed of epithelium surrounded by stroma. Mammary gland postnatal development is an intricate process controlled by steroid and peptide hormones in specific stages that is highly conserved in mammals. Unlike many other organs which undergo development *in utero*, mammary gland development and ductal branching occurs during puberty [1]. While mice have five pairs of mammary glands compared to one pair in humans, mammary gland development remains similar between the two species, making the mouse a good model to study mammary gland development. During embryonic development in the mouse, a rudimentary ductal structure invades the mesenchyma, and remains dormant until approximately 21 days of age, when the onset of ovarian hormone secretion stimulates ductal growth [2].

During puberty, the ovarian hormones estradiol and progesterone stimulate stem cell proliferation in terminal end buds (TEBs) and induce side branching in the mammary gland, respectively [3]. The TEB is found exclusively in the developing mammary gland, and is the main driving force for mammary gland development [4], [5]. The outer layer, or cap layer, of the TEB contains a special population of pluripotent stem cells [2], which ultimately differentiate to form the intermediate, luminal, and myoepithelial cells of the duct [6]. TEB formation and side branching drive mammary gland invasion into the fat pad until the fat pad is filled at approximately 10 weeks of age. Once the ducts have filled the entire mammary fat pad, TEBs are permanently replaced by terminal ducts and alveolar buds [7]. It has been proposed that the adult mammary gland retains a mammary stem cell population which gives rise to epithelial precursor cells [6]. The progeny of the epithelial precursor cells are either ductal precursor cells or alveolar precursor cells. The presence of these stem cells is thought to underlie the ability of the mammary gland to undergo alveolar renewal with subsequent pregnancies [3]. It has also been shown that the mammary epithelium is the major target for carcinogen-induced initiation during tumorigenesis, making the understanding of the mechanisms underlying mammary gland development and function imperative for knowledge of reproductive function and for designing effective anticancer therapies [8].

Protein kinase B/Akt is a serine/threonine kinase that plays a central role in regulation of cell metabolism, cell survival, motility, transcription, and cell-cycle progression [9], [10]. The *Akt* gene family is activated in a phosphoinositide 3 kinase- dependent manner by a variety of signals including cytokines, growth factors, and hormones. The *Akt* family includes three isoforms, *Akt1*, *Akt2*, and *Akt3*, which share 80% amino acid homology but have distinct roles in regulating cellular activity [9]. *Akt1* is the predominant isoform, and plays a role in placental development, organismal growth, adipogenesis, mammary gland lactation and involution, and mammary gland tumorigenesis [11], [12], [13], [14], [15], [16], [17]. *Akt1* ablation has been shown to delay mammary gland growth during pregnancy and lactation, and accelerates the rate of involution following lactation [11], [14]. Conversely, constitutively active *Akt1* delays epithelial cell apoptosis and mammary gland involution [18]. Of note, *Akt1*-deficient dams produce less milk during nursing, and consequently have smaller pup weights regardless of the pup genotype [11], [19]. It has been previously shown that *Akt1* ablation delays the onset of puberty and results in subfertility in C57/Bl6 mice [20]. *Akt1* deficiency also increases the levels of the hormone estradiol and decreases the levels of progesterone at postnatal day 50; however, hormone levels in wild-type and *Akt1*-deficient mice are comparable by postnatal day 90 [20]. Interestingly, estradiol and progesterone are responsible for ductal growth and alveolar expansion in mammary glands, respectively [3]. These previous studies led us to examine the molecular mechanisms underlying the requirement for *Akt1* in mammary gland development and function.

Although *Akt1* is implicated in lactation and involution as well as mammary carcinogenesis, its functional roles in regulating mammary gland development and influencing adult mammary gland structure are not yet fully understood. In this study, we show that *Akt1* is essential for proper mammary gland composition during development and for appropriate gene expression patterns in adulthood. We found that *Akt1* deficiency delayed mammary gland development, with fewer TEBs during puberty and fewer and smaller alveolar buds in adulthood, potentially due to increased apoptosis in the epithelial compartment. Our data further indicate that *Akt1* deficiency resulted in gene expression changes and several altered biological processes, which could affect cellular composition and function, leading to impaired lactation. Specifically, in both adult virgin *Akt1*−/− mammary glands and pseudopregnant *Akt1*−/− glands we found down-regulation of *Btn1a1*, which has previously been shown to be critical for milk lipid secretion [21]. We also show that suppression of *Akt1* in MCF7 human breast epithelial cells decreases expression of *Btn1a1*, indicating a role for *Akt1* in the regulation of *Btn1a1*. Suppression of *Btn1a1* decreased expression of *Btn1a1* but not *Akt1*, suggesting that *Btn1a1* functions downstream of *Akt1*. Taken together, our results indicate that *Akt1* regulates mammary gland postnatal development as well as lobuloalveolar development through the regulation of apoptosis and gene expression.

## Materials and Methods

### Ethics Statement

The Brown University Institutional Animal Care and Use Committee (IACUC) approved all experimental animal protocols in compliance with National Institute of Health guidelines. The IACUC approval number is 0909078 and date of approval was 10/7/2010.

### Animals

*Akt1* heterozygous mice (Mus musculus) on a C57/BL6 background were obtained from the laboratory of Dr. Morris Birnbaum (University of Pennsylvania, Philadelphia, PA). Heterozygous pairs were mated to obtain *Akt1* +/+ and *Akt1* −/− females for experimental procedures. Animals were housed in polycarbonate cages and were given access to water and feed *ad libidum*. Animals were sacrificed by CO_2_ asphyxiation at PND (postnatal day) 11, PND 25, PND 50 or PND 70.

### Mammary gland whole mounts

Inguinal mammary glands from the fourth and ninth positions were removed, fixed in Carnoy's solution for 2–4 hours, stained overnight in carmine solution, rinsed in alcohol and cleared in xylene overnight. Whole mounts were examined and images were taken using a Zeiss Discovery V8 stereomicroscope equipped with an Axiocam MRc camera and Axiovert Software (Carl Zeiss Canada Ltd., Canada). The criterion for scoring varied depending upon the age of the mouse group being evaluated. The primary duct length was measured at PND 11 and PND 25 using the curve spline measurement feature. Since the mammary glands are significantly larger at PND 50 and 70, the length of growth past the lymph node was measured instead of the primary duct length by using the length measurement feature. Ductal outgrowth was measured using the outline spline measurement feature as the area occupied by the entire mammary gland for all time points. Terminal end buds and alveolar buds were measured for all time points in the Zone C area of the mammary gland, which for this study is defined as the area comprised of the outer 1 mm of the entire mammary gland [22]. Terminal end buds were defined as having a diameter of ≥100 µm. The number of total buds was comprised of terminal end buds (≥100 µm) and alveolar buds (<100 µm). The percentage of terminal end buds was calculated by dividing the number of terminal end buds by the number of total buds. The scores from the fourth and ninth position mammary glands from each animal were averaged and the mean was used to represent that animal.

### Histological analysis

Mammary gland whole mounts were manually embedded in paraffin and cut into 7-µm sections for histological analysis. Following deparaffinization, mammary gland sections were washed in 0.1% lithium carbonate to remove carmine aluminum stain. Mammary sections were stained with hematoxylin and eosin (H&E) and martius yellow, soluble blue, brilliant crystal (MSB) stain for fibrin to examine basic pathology. Alveolar bud diameters were measured on the Aperio ImageScope v10.2 (Aperio Technologies Vista, CA). At least 20 measurements per animal were taken and the mean was used to represent that animal.

### Western blots

Thoracic mammary glands were pulverized in liquid nitrogen then lysed in cold RIPA buffer (20 mM Tris 8.0, 150 mM NaCl, 0.5% sodium deoxyocholate, 0.1% SDS, 1% NP-40, 10 mM sodium pyrophosphate, 10 mM sodium fluoride) supplemented with 60 µl/ml HALT protease/phosphatase inhibitors (Thermo Scientific, Waltham, MA) and 1 mM PMSF (Roche, Indianapolis, IN). Lysates were incubated on ice for 20 minutes with frequent vortexing and cleared twice by centrifugation (13,200 rpm, 10 minutes, 4°C). MCF7 cells were lysed in cold lysis buffer (50 mM Tris pH 8.0, 250 mM NaCl, 1% NP-40, 0.1% SDS, 5 mM EDTA, 2 mM sodium orthovanadate, 10 mM sodium pyrophosphate, 10 mM sodium fluoride), incubated on ice for 20 minutes and cleared by centrifugation (13,200 rpm, 10 minutes, 4°C). Protein concentrations were determined using the DC Protein Assay (Bio-Rad). Fifty micrograms of total protein was subjected to SDS-PAGE and transferred onto Immobilon-P PVDF (Millipore, Billerica, MA). Membranes were blocked for 60 minutes at room temperature in 5% non-fat milk/Tris-buffered saline/0.1% Tween (TBST). Membranes were incubated for 2 hours at room temperature then overnight at 4°C in one the following antibodies: AKT1 (#2938, 1∶1000 in 5% BSA/TBST), AKT2 (#2964, 1∶1000 in 5% BSA/TBST), AKT3 (#3788, 1∶1000 in 5% BSA/TBST), or GAPDH (AM4300, 1∶4000, Applied Biosystems). All antibodies were obtained from Cell Signaling Technology (Danvers, MA) unless indicated otherwise. Membranes were washed 3 times (5 minutes per wash) in TBST, and incubated for 60 minutes at room temperature in horseradish peroxidase conjugated goat anti-rabbit IgG (1∶2000, Cell Signaling) or goat anti-mouse IgG (1∶10,000 Millipore) diluted in 5% milk/TBST. Membranes were washed 3 times (5 minutes per wash) in TBST and once in TBS prior to visualization using enhanced chemiluminescence (Thermo Scientific).

### Determination of apoptotic rates by terminal deoxynucleotidyl transferase mediated dUTP nick end labeling (TUNEL) staining and quantitation

PND 70 mammary glands were stained for apoptosis using the ApopTag kit (Chemicon, Temecula, CA) according to the manufacturer's instructions. Tissues were counterstained with hematoxylin. Digital images were captured using the Aperio Scanscope Imagine System (Aperio Technologies, Vista, CA), and TUNEL positive nuclei were measured using the nuclear algorithm analysis on the Aperio Imagescope v10.2. At least 200 cells per animal were measured.

### RNA isolation

RNA was isolated from thoracic mammary glands from mice (PND70) previously flash frozen in liquid nitrogen or MCF7 cells using the mirVANA RNA Isolation Kit (Ambion Inc., Austin, TX) according to manufacturer's protocols. RNA concentration was quantified using a ND-1000 spectrophotometer (Nanodrop, Wilmington, DE), aliquoted, and stored at −80°C until needed.

### RNA microarray hybridization

Integrity of RNA isolated from thoracic mammary glands was assessed using an Agilent 2100 Bioanalyzer (Agilent Technologies, Palo Alto, CA). Complementary DNA (cDNA) was synthesized from 300 ng of total RNA and purified according to the manufacturer's protocol (Affymetrix, Santa Clara, CA.). Equal amounts of purified cDNA were used as the template for subsequent *in vitro* transcription reactions for complementary RNA (cRNA) amplification and biotin labeling using the Affymetrix RNA Transcript Labeling Kit. cRNA was purified and fragmented according to the protocol provided with the GeneChip Sample Cleanup Module (Affymetrix, Santa Clara, CA). All GeneChip microarrays (Mouse Genome 430 2.0 Array) were hybridized, washed, stained, and scanned using the Complete Gene Chip Instrument System according to the Affymetrix Technical Manual. The Mouse Genome 430 2.0 microarray contained 17 probe sets.

### Microarray data analysis

All data is MIAME compliant and data is publically available at GEO with accession number: GSE30671. Affymetrix CEL files were imported into Partek® Genomics Suite™ 6.5 (Partek Inc., St. Louis, MO) using the default Partek normalization parameters. The robust multiarray average (RMA) normalization method was used to normalize data. Statistically significant differentially expressed genes between *Akt1*+/+ and *Akt1*−/− mammary glands were defined as having a p<0.05 as determined by a one-way analysis of variance (ANOVA). The recovered P-values of the statistically significant genes were corrected using a step-up false discovery rate value of 5%. The final list of differentially expressed genes included only those genes with a 1.5-fold change (up or down) with a p<0.05. Data was further analyzed using Ingenuity Pathways Analysis (IPA) (Ingenuity® Systems, www.ingenuity.com). The IPA Functional Analysis program was used to identify the biological functions and/or diseases that were most significant to the data set. Only molecules from the dataset meeting the p value cutoff of 0.05 and were associated with biological functions and/or diseases in Ingenuity's Knowledge Base were considered for the analysis. Right-tailed Fisher's exact test was used to calculate a p-value determining the probability that each biological function and/or disease assigned to that data set was due to chance alone. IPA's Pathway Analysis generated graphical representation of the molecular relationships between molecules, represented as nodes, and the biological relationship between nodes, represented as an edge (line). All edges were supported by at least 1 reference from the literature or from canonical information stored in the Ingenuity Pathways Knowledge Base. Nodes are displayed using various shapes that represent the functional class of the gene product, while the intensity of the node color indicates the degree of up- (red) or down- (green) regulation. Edges are displayed with labels describing the nature of the relationship between the nodes.

### Quantitative RT-PCR

Total RNA (1 µg) was treated with DNase (Promega, Madison, WI) and reverse-transcribed using the iScript cDNA Synthesis Kit (Bio-Rad) according to the manufacturer's protocols. cDNA templates were amplified with pre-optimized mouse-specific QuantiTect® primer assays (Qiagen, Valencia, CA) using iQ SYBR Green Supermix (Bio-Rad) on an iCycler iQ Multicolor Real-time PCR Detection System (Bio-Rad). Each 25 µl sample was run in triplicate, and mRNA levels were analyzed relative to hypoxanthine phosphoribosyltransferase (HPRT). Log2-transformed relative expression ratios were calculated as described using the equation set forth by Pfaffl (Pfaffl 2001).

### Pseudopregnancy stimulation

Female mice at 60–90 days old were injected intraperitoneally (i.p.) with 5.0 international units (IU) of PMSG (Sigma Aldrich) followed by 5.0 IU of hCG i.p. 48 hours later. Mammary glands were excised from mice 18 hours after hCG administration, thoracic glands were flash frozen for RNA, and inguinal glands were either processed for whole mounts or fixed in 10% neutral buffered formalin.

### Immunofluorescence

Indirect immunofluorescence was performed on formalin-fixed, paraffin-embedded 7 µm sections. Sections were re-hydrated, and antigen retrieval was performed by heating slides in 10 mM sodium citrate in a steamer for 20 minutes. Sections were blocked overnight with normal goat and rabbit serum (Vector Laboratories, Burlingame, CA), and incubated with primary antibodies against Btn1a1 (AV45164, Sigma-Aldrich) and Akt1 (sc-1618, Santa Cruz) followed by Alexa Fluor 488 rabbit anti-goat (Invitrogen) and Alexa Fluor 594 goat anti-rabbit (Invitrogen). Sections were counterstained with DAPI and visualized on a microscope equipped with a digital camera and software.

### Cell culture and short-hairpin RNA knockdown

MCF-7 cells, a generous gift from the lab of Dr. Edward Filardo (Brown University), were cultured in phenol red-free DMEM/F-12 (Hyclone), 10% fetal bovine serum, 0.1% penicillin/streptomycin in a humidified atmosphere (5% CO_2_/95%air). Retroviral-mediated shRNA knockdown was performed as described in [23]. The oligonucleotides containing targeting sequences (shown in uppercase) for firefly luciferase [short-hairpin luciferase (shLuc)] controls, human Akt1 (shAkt1), and human Btn1a1 (shBtn1a1) were asfollows: 5′gatccccGCGACCAACGCCTTGATTGttcaagagaCAATCAAGGCGTTGGTCGCtttttgga-3′ for luciferase, 5′-gatccccGAGTTTGAGTACCTGAAGCttcaagagaGCTTCAGGTACTCAAACTCtttttggaaa-3′ for Akt1, and 5′-gatccccgcgt GatagaggagagaatttcaagagaATTCTCTCCTCTATCACGCttttggaaa-3′ for Btn1a1. Knockdown cells were maintained in 1.0 µg/ml puromycin. For cell proliferation 100,000 cells were plated in each well of a 6-well plate. Cell proliferation was determined by Trypan blue exclusion using a TC-10 automated cell counter (Bio-Rad, Hercules, CA), and calculating the percent change of in number of cells relative to cell number on day 0.

### Statistical analysis

A Bartlett test for homogeneity followed by a two-tailed students t-test adjusted for equal variance were performed to test for statistical significance. For genotype and exposure interactions, a two-way ANOVA followed by a Tukey HSD test was performed. A P value of <0.05 was considered to be statistically significant. Statistics were calculated using R software (R Project).

## Results

### *Akt1* deficiency delays mammary gland postnatal development

**Figure 1:**
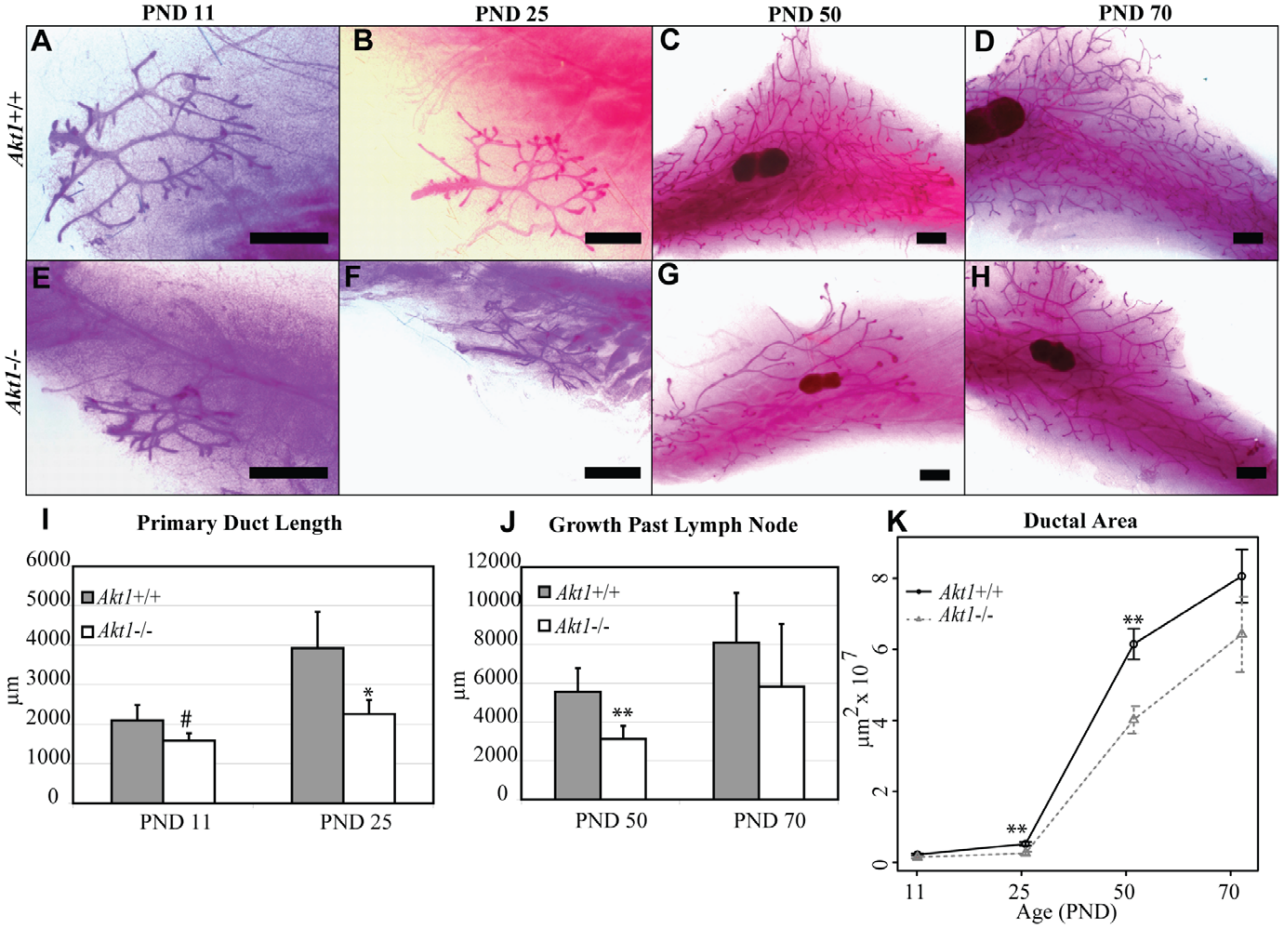
*Akt1* promotes postnatal mammary gland growth. Representative whole mounts of *Akt1*+/+ (A–D) and *Akt1*−/− (E–H) mammary glands at PND 11 (A,E), PND 25 (B,F), PND 50 (C,G), and PND 70 (D,H). Measurement of primary duct length at PND 11 and PND 25 in *Akt1*+/+ and *Akt1*−/− glands (I). Measurement of the growth past the lymph node at PND 50 and PND 70 in *Akt1*+/+ and *Akt1*−/− glands (J). Quantification of ductal outgrowth for *Akt1*+/+ and *Akt1*−/− mammary glands at PND 11, 25, 50, and 70 (K). Data represented in line plot as mean +/− standard error. Data represented in bar plots as mean + standard error. Scale bars indicate 1,000 µm. Statistical analyses were conducted using a two-tailed student's t test. **p<0.01, *p<0.05. n = 3–8 per genotype, per age group.

Previously identified roles for *Akt1* in developmental delay and reproductive impairment [11], [14], [17], [19], [20] led us to examine its involvement in postnatal mammary gland development. We first examined the growth patterns of mammary glands in *Akt1*+/+ and *Akt1*−/− mice at different stages of postnatal development, including neonatal (PND 11), juvenile (PND 25), pubertal (PND 50), and adult (PND 70) stages (Fig. 1 A–H). *Akt1*−/− mammary glands exhibited significantly stunted ductal outgrowth during the juvenile and pubertal time periods (PND 25 and PND 50, respectively), but by adulthood (PND 70) the glands occupied the same amount of space in the fat pad compared to *Akt1*+/+ mice (Fig. 1 K). Accordingly, primary duct length was reduced in *Akt1*−/− glands at both PND 11 and PND 25 (Fig. 1 I). Analogous to the ductal outgrowth, at PND 50 growth past the lymph node was shorter in *Akt1*−/− glands (Fig. 1 J). At adulthood, the growth past the lymph node was comparable in *Akt1*−/− and *Akt1*+/+ glands (Fig. 1 J). Overall, these results suggest that *Akt1* is not necessary for the initial formation of the rudimentary ductal tree during the neonatal period, but that it does mediate ductal growth during puberty until adulthood when *Akt1*−/− glands reach full outgrowth similar to their wild-type counterparts.

### *Akt1* regulates the cell population in developing and adult mammary glands

**Figure 2:**
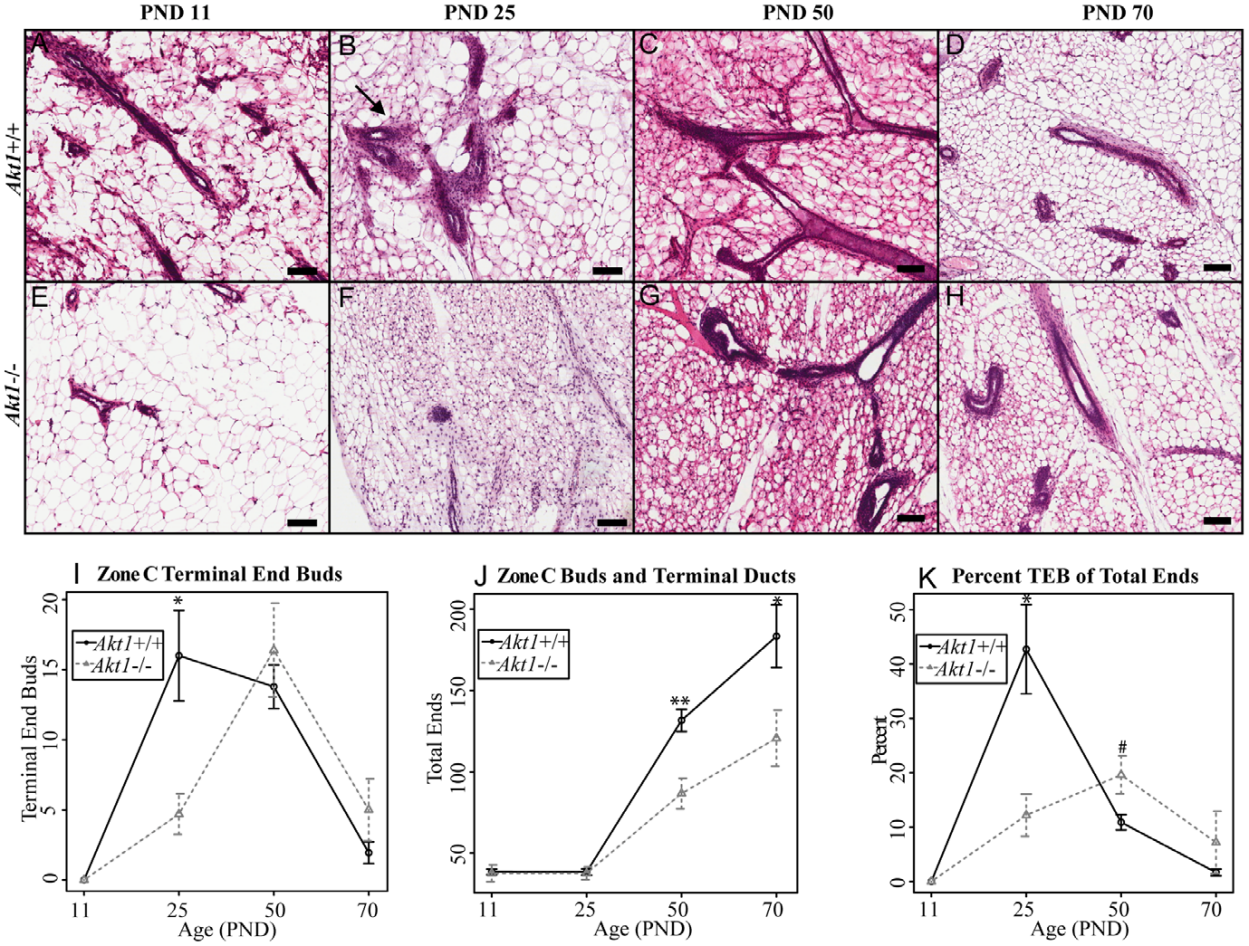
*Akt1* mediates the terminal end bud, terminal duct and alveolar bud population. Representative H&E images of *Akt1*+/+ (A–D) and *Akt1*−/− (E–H) mammary glands at PND 11 (A,E), PND 25 (B,F), PND 50 (C,G), and PND 70 (D,H). Arrow indicates terminal end buds (B) Quantification of terminal end buds in *Akt1*+/+ and *Akt1*−/− mammary glands at PND 11, 25, 50 and 70 (I). Quantification of total buds and terminal ducts in *Akt1*+/+ and *Akt1*−/− mammary glands at PND 11, 25, 50 and 70 (J). Quantification of the terminal end buds relative to the total number of buds of *Akt1*+/+ and *Akt1*−/− mammary glands at PND 11, 25, 50 and 70 (K). Scale bars indicate 100 µm. Data represented in line plots as mean +/− standard error. Statistical analyses were conducted using a two-tailed student's t test. **p<0.01, *p<0.05, #p<0.1. n = 3–8 per genotype, per age group.

Considering the effects that *Akt1* deficiency elicited on ductal growth in the mammary gland, we next investigated the effects of *Akt1* loss on ductal branching and alveolar expansion in the developing gland. Initial results indicated the *Akt1* deficiency had little effect on initial side branching at either PND 11 or PND 25 (Fig. S1). We evaluated terminal end bud bifurcation by quantifying the number of terminal end buds, alveolar buds, and terminal ducts in the Zone C area of the mammary gland throughout development (Fig. 2, A–H) [22]. *Akt1* deficiency decreased the number of terminal end buds during the juvenile time period (Fig. 2 I). The terminal end bud is characterized by having an inner layer of body cells surrounded by an outer layer of cap cells, which give rise to luminal cells and basal cells, respectively [3]. This outer layer of cap cells is evident in the *Akt1*+/+ mammary glands during puberty, but is near absent in the *Akt1*−/− glands (Fig. 2 B, F). These terminal end buds are normally present during puberty, and are permanently replaced with alveolar buds and ducts when the mammary gland reaches the end of the fat pad [7]. Therefore, calculating the percentage of terminal end buds is useful for gauging the relative stage of development of the mammary gland. Although *Akt1*−/− glands contained a lower percentage of TEBs at PND25, there was a trend for a higher percentage of TEBs during puberty (PND 50), suggesting a more immature phenotype for the *Akt1*−/− glands at puberty (Fig. 2 K).

*Akt1* deficiency also decreased the total number of alveolar buds and ducts during puberty (PND 50), which persisted into adulthood (PND 70) (Fig. 2 J). While *Akt1*−/− glands eventually reach the same size as their wild-type counterparts, the overall alveolar bud and terminal duct population remains lower, indicating a permanent change in mammary gland structure. This alveolar bud deficiency could be attributed to the earlier lack of terminal end buds, which contain the progenitor cells for alveolar and ductal myoepithelial and epithelial cells. Importantly, this apparent lack of alveolar buds and ducts may be a potential reason for abnormal mammary gland reconstruction during pregnancy and lactation in *Akt1*−/− glands as seen in other studies [14].

### *Akt1* influences mRNA patterns in the adult mammary gland

**Figure 3:**
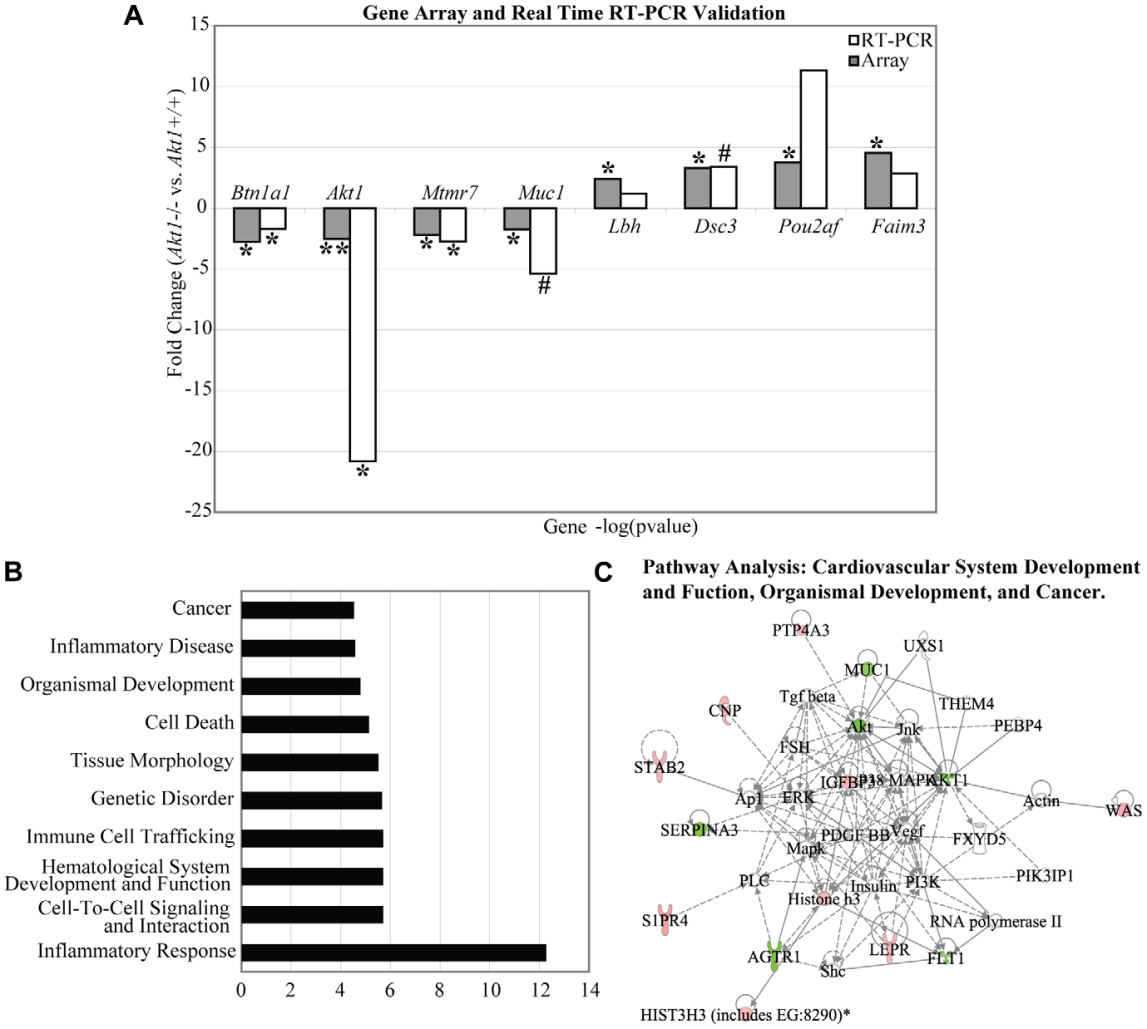
*Akt1* influences gene expression patterns in the adult mammary gland. Microarray results were validated with qRT-PCR for the genes *Btn1a*, *Akt1*, *Mtmr7*, *Muc1*, *Lbh*, *Dsc3*, *Pou2af*, and *Faim3*. Data represented as fold change in gene expression for *Akt1*−/− vs. *Akt1*+/+ (A). IPA analysis generated the top ten biological functions and diseases altered by the loss of *Akt1* (B) IPA analysis generated an association network connecting cardiovascular system development and function, organismal development, and cancer (C). Statistical analyses for qRT-PCR were conducted using a two-tailed student's t test. **p<0.01, *p<0.05, #p<0.1. n = 3 per genotype for microarray, n = 6 per genotype for qRT-PCR.

**Figure 4:**
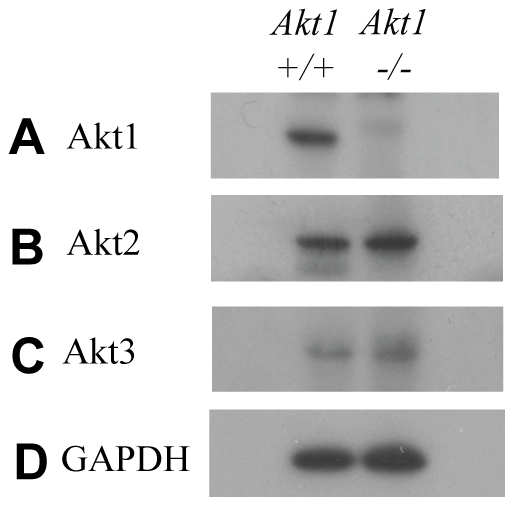
Western blot analysis of Akt isoform expression in PND 70 *Akt1*+/+ and *Akt1/−* mammary glands. Western blots of Akt1 (A), Akt2 (B), Akt3 (C), and GAPDH (D) in PND 70 Akt1+/+ and Akt1−/− mammary glands are representative of 5–8 animals per genotype.

The deficiency in alveolar buds and terminal ducts in *Akt1*−/− adult mammary glands led us to examine the effects of *Akt1* deficiency on gene expression in the adult mammary gland. We performed Affymetrix gene arrays on PND 70 *Akt1*+/+ and *Akt1*−/− thoracic mammary glands, which indicated differential expression of 119 genes with a fold change of <1.5 and a p-value of <0.05 (Table S1). Real-time RT-PCR validation of selected genes from the gene array indicated strong correlation between gene array results and RT-PCR. The genes chosen for validation were *butyrophilin*, *subfamily 1*, *member A1* (*Btn1a1*), *v-akt murine thymoma viral oncogene homolog 1* (*Akt1*), *myotubularin related protein 7* (*Mtmr7*), *mucin 1* (*Muc1*), *limb bud and heart development homolog* (*Lbh*), *desmocollin 3* (*Dsc3*), *POU class 2 associating factor 1* (*Pou2af*), and *fas apoptotic inhibitory molecule 3* (*Faim3*) (Fig. 3 A). We also validated the absence of Akt1 protein in the adult mammary glands by western blot, as well as the presence of Akt isoforms Akt2 and Akt3 (Fig. 4).

Ingenuity pathway analysis indicated several altered biological functions as a result of *Akt1* ablation, including cell-to-cell signaling, intercellular interaction, tissue morphology, organismal development, and cancer within the top ten altered functions (Fig. 3 B). Pathway analysis further illustrated that the pattern of differential gene expression was highly associated with cardiovascular system development and function, organismal development, and carcinogenesis pathways (Fig. 3 C).

### Alveolar cell death incidence and alveolar bud diameter are dependent on *Akt1*

**Figure 5:**
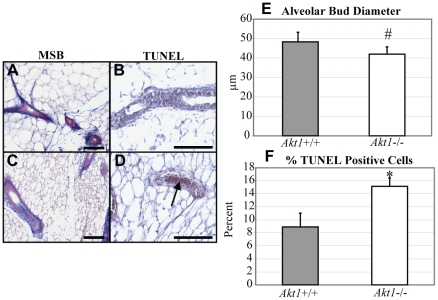
*Akt1* influences alveolar bud diameter and apoptosis incidence in adult mammary glands. Martius yellow, soluble blue, brilliant crystal (MSB) stain of *Akt1*+/+ (A) and *Akt1*−/− (C) PND 70 mammary glands. Blue staining indicates collagen and purple staining indicates fibrin. TUNEL staining in *Akt1*+/+ (B) and *Akt1*−/− (D) PND 70 mammary glands. Measurement of alveolar bud diameter in *Akt1*+/+ and *Akt1*−/− PND 70 mammary glands (E). Measurement of TUNEL positivity in *Akt1*+/+ and *Akt1*−/− PND 70 mammary glands (F). Arrow indicates apoptotic cells in the *Akt1*−/− gland. Scale bars indicate 100 µm. Data represented as mean plus standard error. Statistical analyses were conducted using a two-tailed student's t test. #p = 0.08, *p<0.05. n = 4 per genotype for (E), n = 3 per genotype for (F).

Our previous results indicated that *Akt1* is necessary to maintain proper alveolar bud population in adulthood. Given the pro-survival role of *Akt1* during pregnancy and lactation [14], [19] and the ability of *Akt1* to block several pro-apoptotic processes, we examined the effect of *Akt1*−/− loss on localization of fibrin and collagen in the mammary gland, alveolar bud size, and mammary epithelial cell death. Collagen and fibrin deposition were not altered by the ablation of *Akt1*, indicating normal stromal architecture in *Akt1*+/+ and *Akt1*−/− mammary glands (Fig. 5 A, C). However, there was a strong trend for decreased alveolar bud diameter in *Akt1*−/− mammary glands, concomitant with increased epithelial cell apoptosis (Fig. 5 B, D–F). Considering that our gene array results denoted cell death and tissue morphology to be two of the top ten altered biological processes altered by *Akt1* deficiency, these data further suggest that apoptosis and alveolar bud formation in the mammary gland are dependent, in part, on *Akt1*.

### *Akt1* affects mammary gland lobuloalveolar development and *Btn1a1* expression during psuedopregnancy

**Figure 6:**
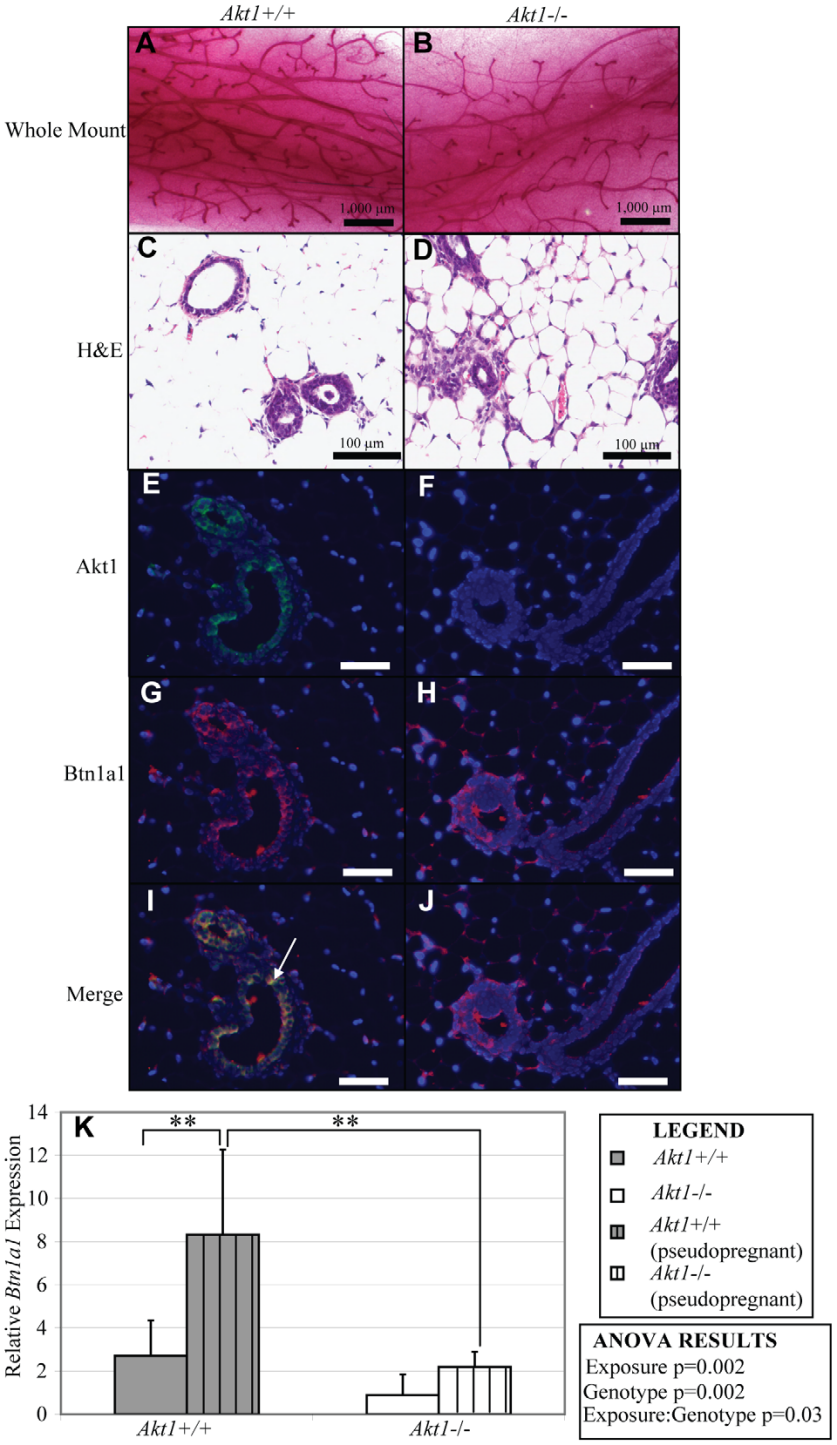
*Akt1* is necessary for *Btn1a1* expression following pseudopregnant hormonal stimulation. Representative whole mounts of pseudopregnant *Akt1*+/+ (A) and *Akt1*−/− (B) mammary glands, and H&E stained (C, D) mammary glands. Representative immunofluorescence of Akt1 and Btn1a1 in pseudopregnant *Akt1*+/+ (E, G, I) and *Akt1*−/− (F, H, J) mammary glands. Arrow indicates co-expression of Akt1 and Btn1a1 the in *Akt1*+/+ mammary gland epithelium. *Btn1a1* gene expression as determined by real time RT-PCR in virgin and pseuodopregnant *Akt1*+/+ and *Akt1*−/− thoracic mammary glands (K). Exposure = pseudopregnancy. Scale bars for whole mounts and histology/immunohistochemistry indicate 1,000 µm and 100 µm, respectively. Data represented as mean plus standard error. Statistical analyses were conducted by a two-way ANOVA followed by a Tukey HSD test. **p<0.01 for Tukey HSD post-hoc test. n = 3–6 per genotype, per exposure.

Prior studies have indicated that *Akt1* is necessary for proper lobuloalveologenesis and lactation during pregnancy and nursing. We chose to study the role of *Akt1* in alveolar differentiation during pseudopregnancy based on our findings of its effects on mammary gland development during puberty and on subfertility in *Akt1*−/− females. Similar to previous work using naturally pregnant mice, *Akt1* affects the frequency of formation of lobuloalveolar structures in pseudopregnant mice (Fig. 6 A–D). While *Akt1* deficiency reduces the number of differentiated alveolar buds, it is not required for alveolar differentiation, as indicated by the presence of alveoli (Fig. 6 D). This is in contrast to previous work that concluded that ablation of *Akt1* inhibits the formation of lobuloalveolar structures, but this is likely due to the fact that our study induced pseudopregnancy, whereas prior work utilized naturally pregnant animals [14]. Considering the decreased number of alveolar buds and ducts in adult virgin *Akt1*−/− mammary glands, our results indicate that *Akt1* is necessary for the initial formation of alveolar buds during development and relative number of lobuloalveolar structures formed during pregnancy, but is not necessary for alveologenesis.

Our gene array and real-time RT-PCR results indicated that the loss of *Akt1* in the mammary gland significantly decreased expression of *Btn1a1*, a member of the butyrophilin Ig superfamily that is basally expressed in the brain, heart, lymph node, ovary and uterus, thymus, lung, spleen, and mammary gland [24]. *Bnt1a1* is highly expressed in the secretory epithelium of lactating mammary glands, and is essential for milk lipid secretion [21]. Btn1a1 and Akt1 were co-expressed in pseudopregnant mammary glands (Fig. 6 E, G, I), and expression of Btn1a1 was decreased in *Akt1*−/− glands, signifying that *Akt1* is necessary for proper protein expression of Btn1a1 in the mammary gland (Fig. 6 H). Additionally, while *Akt1*+/+ mammary glands responded to pseudopregnancy by significantly increasing the levels of *Btn1a1* as compared to virgin glands, *Akt1*−/− mammary glands failed to do so (Fig. 6 K). *Btn1a1* expression in *Akt1*−/− glands during pseudopregnancy was also significantly decreased as compared to pseuodpregnant *Akt1*+/+ glands. A two-way ANOVA analysis revealed that both *Akt1* genotype status and exposure (pseudopregnancy) were significant factors in determining *Btn1a1* expression.

### *Akt1* promotes cell proliferation and *Btn1a1* and *ß-casein* gene expression in MCF7 human breast epithelial cells

**Figure 7:**
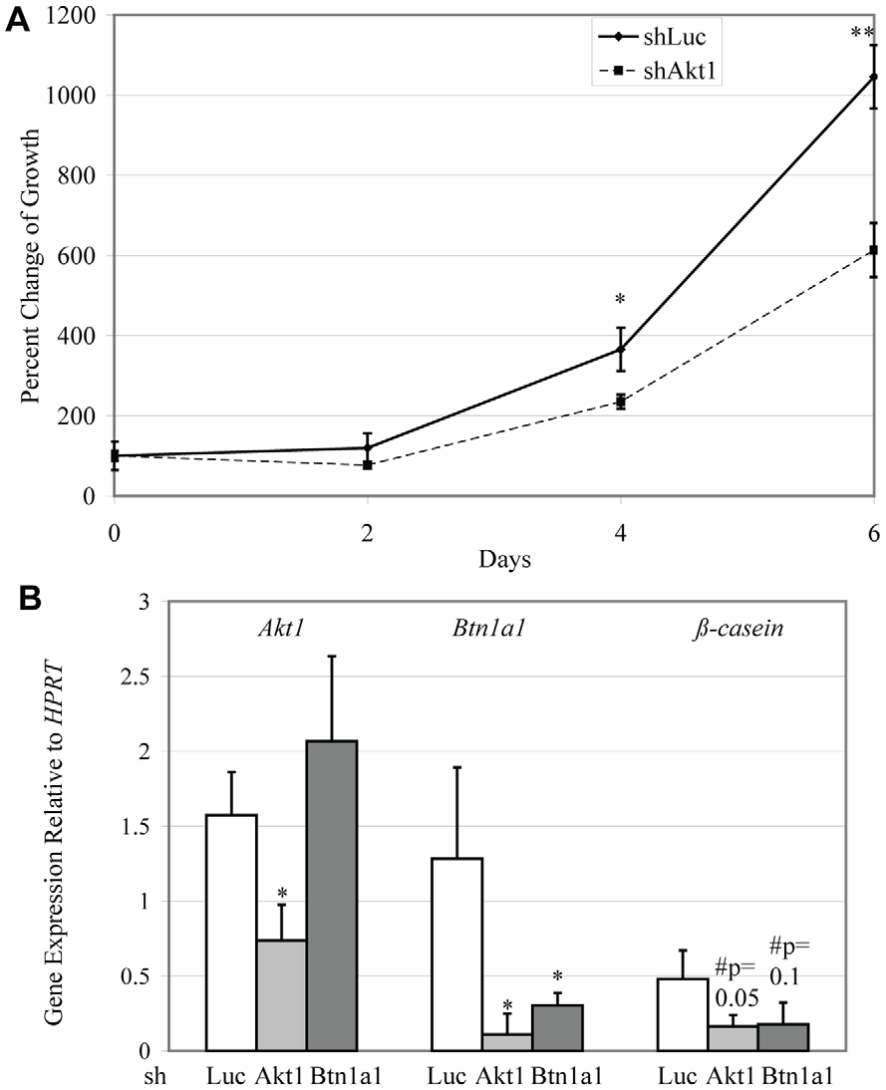
*Akt1* is required for epithelial cell proliferation and *Btn1a1* and *ß-casein* gene expression. Percent change in cell growth over 6 days in MCF7 cells expressing shAkt1 or shLuciferase control (A). Relative gene expression of *Akt1*, *Btn1a1*, and *ß-casein* in shAkt1, shBtn1a1, or shLuciferase cells (B). Data represented as mean plus standard error. Statistical analyses were conducted using two-tailed student's t tests. #p<0.1, **p<0.01, *p<0.05 compared to shLuciferase control. n = 3 independent infections.

In order to directly address whether *Btn1a1* or *ß-casein* are downstream of Akt1, we evaluated the effect of shRNA-mediated knockdown of *Akt1* or *Btn1a1* in MCF7 human breast epithelial cells on *ß-casein*, a gene that encodes for a milk protein expressed during early lactational mammary epithelial differentiation [11]. MCF7 cells expressing shRNA targeting Akt1 (shAkt1) exhibited significant decreases in cell proliferation after 4–6 days of growth in culture (Fig. 7 A). Knockdown of Akt1 also resulted in significant decreases in both *Btn1a1* and *ß-casein* expression (Fig. 7 B), while knockdown of *Btn1a1* (shBTN1A1) decreased expression of *Btn1a1* and *ß-casein*, but not *Akt1* (Fig. 7 B, Fig. S2.).

## Discussion

The mammary gland is a critical reproductive organ, and the abilities of the adult mammary gland to undergo normal development and proper lobuloalveolar remodeling during pregnancy are critical for reproductive health. The mammary gland is distinct from other organs because it undergoes most of its branching postnatally with the onset of puberty rather than *in utero*. Proper branching morphogenesis is fundamental for functional development and differentiation to occur in adulthood. Using an *Akt1*+/+ and *Akt1*−/− mouse model to examine postnatal development *in vivo*, we found that *Akt1* is essential for proper mammary gland organogenesis and appropriate adult mammary homeostasis. Mammary gland development can be separated into three distinct stages: embryonic, adolescent, and adult. We found that the initial branching and formation of the rudimentary duct occurred normally in *Akt1*−/− mammary glands, indicating that embryonic mammary gland development is *Akt1*-independent. These results are not surprising considering embryonic mammary development is also hormone-independent, and PI3K/AKT activates ERα [25].

We did find that postnatal mammary gland development is *Akt1-*dependent. Unlike embryonic growth, postnatal development requires estrogen and progesterone, which are secreted by the ovaries at approximately three weeks of age in the mouse. Ductal outgrowth of inguinal mammary glands was significantly reduced in *Akt1*−/− glands during early and late pubertal time points. However, by adulthood these glands occupied the same amount of space in the fat pad as their wild-type counterparts. Our previous work indicated that *Akt1*−/− female mice had higher levels of estradiol and lower levels of progesterone than *Akt1*+/+ females during late puberty, but that these levels were similar in both genotypes by adulthood irrespective of *Akt1* status [20]. Considering that postnatal ductal growth and alveolar expansion are dependent on estradiol and progesterone, the higher levels of progesterone most likely have a compensatory effect on ductal outgrowth, allowing for the *Akt1*−/− mammary glands to reach full size. Conversely, the levels of estradiol may be a contributing factor to the altered terminal end bud and alveolar expansion seen in the *Akt1*−/− glands. Mammary gland postnatal ductal development is driven by the rapid growth of the terminal end bud, a unique structure that contains dichotomously dividing stem cells during puberty. The terminal end bud contains a ‘tube within a tube’ structure, with an outer layer of undifferentiated cap cells and an inner layer of luminal epithelial cells. Both layers have high rates of mitosis, and normally have rates of forward growth through the fat pad of about 0.5 mm per day [26]. *Akt1* deficiency resulted in a significant decrease in the terminal end bud population during puberty, and a significant decrease in total alveolar buds and a strong trend for decreased alveolar bud size in adulthood. We also demonstrated that suppression of *Akt1* in human breast epithelial cells inhibited cell proliferation, further illustrating the importance of *Akt1* in mammary epithelial cell growth. The growth of the terminal end bud relies both on estradiol and growth factors elicited by the stroma, such as insulin-like growth factor-1 (IGF-1). Notably, the AKT pathway has been implicated in both estrogen and insulin signaling [9], [25]. Moreover, Akt has been shown to be phosphorylated downstream of IGF-1 [27]. In our model, *Akt1* could be a potential mediator of IGF-1 and estrogen signaling to the terminal end bud, and its deficiency results in a lack of bifurcation.

The permanent structural changes in *Akt1*−/− mammary glands led us to examine alterations in gene expression in adult glands. We chose to validate a set of genes based on their high rates of fold change and relative importance in growth and differentiation processes. For example, *Akt1*−/− glands exhibited increased expression of *Lbh*, a gene normally expressed during development in branched limbs and the heart, which could be a compensatory mechanism for the observed deficiency in postnatal growth and bud development. Over-expression and deregulation of *Lbh* results in suppression of epithelial cell differentiation and a potential involvement in Wnt-induced breast tumorigenesis [28]. Our findings further suggest that the lack of *Akt1* in the mammary gland may also disrupt its functional role in lactation. *Akt1* has previously been found to be involved in the proper formation of lobuloalveolar units during pregnancy, as well as in proper milk production during nursing. The phenotype of *Akt1*−/− mammary glands has been described, including the inhibition of lobuloalveolar units and survival in mammary epithelia during pregnancy [14].

We chose to investigate the molecular basis of abnormal mammary gland function based on our prior results indicating decreased expression of *Btn1a1* in *Akt1*−/− mammary glands. *Btn1a1* has recently been found to be a crucial gene for proper milk lipid secretion during lactation. We induced pseuodopregnacy to examine the influence of *Akt1* on *Bnt1a1* during pregnancy by PMSG and hCG injections in *Akt1*+/+ and *Akt1*−/− adult females since *Akt1*−/− females have high rates of fetal resorptions and are subfertile [20]. Following hormonal stimulation, *Akt1*+/+ mammary glands exhibited increased expression of *Btn1a1*, suggesting that *Btn1a1* induction occurs normally during pregnancy, likely in preparation for milk production during nursing, as *Btn1a1* is required for the secretion of milk lipid droplets [24]. *Akt1*−/− mammary glands failed to induce an increase in expression of *Btn1a1* during pseudopregnancy compared to a virgin state, and had significantly decreased *Btn1a1* expression compared to pseuodopregnant *Akt1*+/+ glands. Furthermore, knockdown of *Akt1* in human breast epithelial cell decreased expression of *ß-casein*, *Btn1a1* and *Akt1*, and knockdown of *Btn1a1* decreased expression of *ß-casein* and *Btn1a1*, but not *Akt1*. These data suggest that *Btn1a1* is downstream of *Akt1*, and *ß-casein* is further downstream. These data, taken together with the previously shown roles of *Btn1a1* and *Akt1* in lactation, suggest a regulation of *Btn1a1* by *Akt1* in the mammary gland, and that *Akt1* may promote milk protein production through *Btn1a1* and subsequent *ß-casein* expression. Collectively, while *Akt1*−/− mammary glands can form alveoli during pseudopregnancy, they are fewer in number and potentially dysfunctional in their milk production.

IPA revealed that several genes involved in organismal development, cell death, tissue morphology, and cancer were altered *Akt1*−/− mammary glands. The roles of the PI3K/AKT pathway in cell survival and apoptosis have been extensively studied, but we found differential expression of additional genes that may be involved in this process, including *Mtmr7*, *Pou2af*, and *Faim3*, which play roles in either cell survival or apoptosis. We also found that *Akt1* deficiency resulted in decreased alveolar bud diameter and increased epithelial cell apoptosis, which could decrease the quantity of alveolar buds and terminal ducts in adult *Akt1*−/− glands. However, cell growth in terminal end buds and alveolar buds not only relies on mitogenic signals for proliferation, but also on cell-cell contacts for forward growth. Cell-cell signaling and interaction was another biological process altered by *Akt1* deficiency, which included several altered genes such as *Dsc3*, a member of the cadherin superfamily. Cadherins are calcium-dependent cell-adhesion proteins that mediate cell-cell interaction, and several cadherins are expressed in mammary gland alveolar buds and terminal end buds. Previous research has indicated that disruption of cell-cell contacts inhibits forward growth, suggesting that these interactions are essential for proper mammary gland growth through the fat pad [26]. It is possible that aberrant signaling in the absence of *Akt1* disrupted cell-cell signaling, resulting in improper growth, as demonstrated in the delayed ductal outgrowth and deficiency in terminal end bud growth and alveolar bud development. Collectively, our findings suggest that *Akt* signaling is essential for proper alveolar bud apoptosis, quantity, growth, and size.

Considering prior studies and our novel findings, it is likely that *Akt1* is necessary for both proper mammary gland postnatal growth and functional adult development. Regarding postnatal growth, *Akt1* appears essential for ductal outgrowth through the fat pad as well as for the bifurcation of terminal end buds and formation of alveolar buds and terminal ducts. Permanent changes in mammary gland composition and gene expression are likely contributing factors to the abnormal function of adult *Akt1*−/− mammary glands. Regarding *Btn1a1*, it seems that *Akt1* is essential for its expression both during a basal virgin state as well as during pseudopregnancy. The lack of *Btn1a1* and *Akt1* expression, both of which influence milk lipid production, can result in increased offspring mortality, suggesting these genes are essential for proper reproductive health and offspring growth.

## Supporting Information

Figure S1 ***Akt1*** **does not mediate initial side branching in the developing mammary gland.** Quantification of the number of branch points in the mammary gland at PND 11 and PND 25. Data represented as mean plus standard deviation. Statistical analyses were conducted using a two-tailed student's t test. n = 3–8 per genotype, per age group.
(TIF)

Figure S2 **Selective knockdown of the Akt1 isoform in MCF7 human mammary epithelial cells.** Western blot analysis of Akt1, Akt2, Akt3, and GAPDH in MCF7 cells expressing shLuciferase, shBtn1a1 or shAkt1.
(TIF)

Table S1 **Differentially expressed gene list in** ***Akt1−/−*** **mammary glands.** Listed are the statistically significant genes with a p<0.05 and a fold-change of <1.5 in adult *Akt1*−/− mammary glands compared to *Akt1*+/+ mammary glands.
(DOC)
