# Supplementary figures and images for: Akt1 Is Essential for Postnatal Mammary Gland Development, Function, and the Expression of Btn1a1
Source: PLoS One. 2011 Sep 7;6(9):e24432. doi: 10.1371/journal.pone.0024432 (PMC3168520; doi:10.1371/journal.pone.0024432)

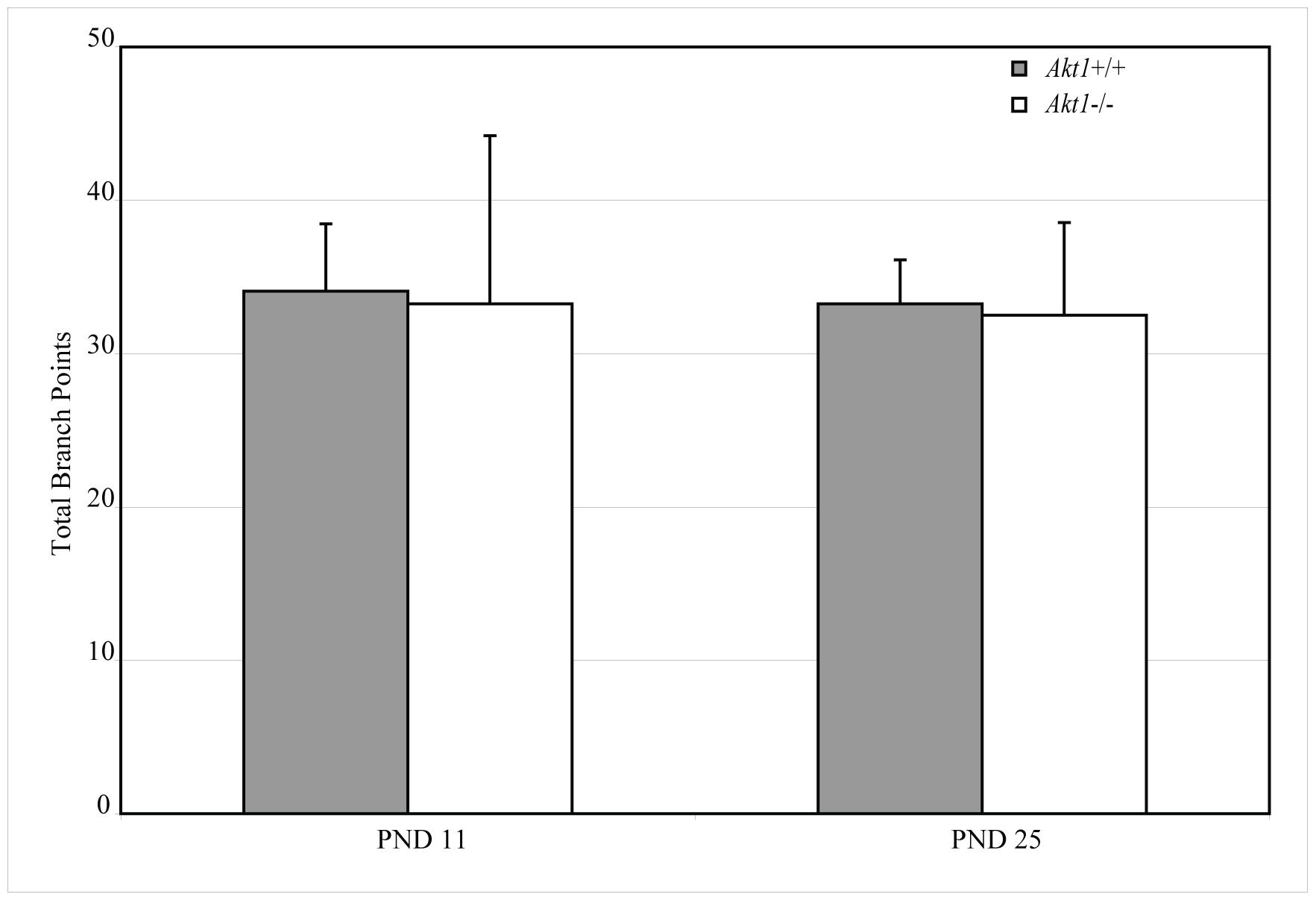

Supplement: Figure S1 — Akt1 does not mediate initial side branching in the developing mammary gland. Quantification of the number of branch points in the mammary gland at PND 11 and PND 25. Data represented as mean plus standard deviation. Statistical analyses were conducted using a two-tailed student's t test. n = 3–8 per genotype, per age group. (TIF) [file pone.0024432.s001.tif]

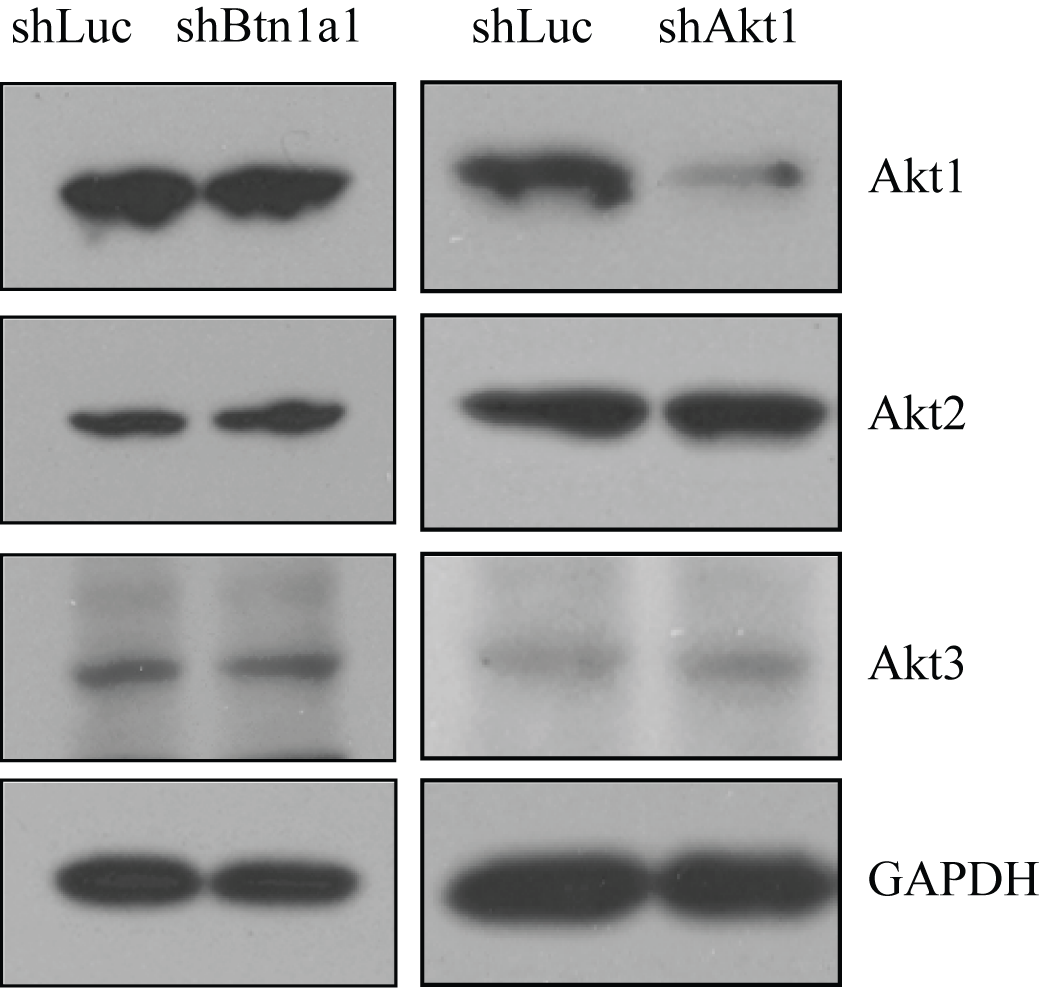

Supplement: Figure S2 — Selective knockdown of the Akt1 isoform in MCF7 human mammary epithelial cells. Western blot analysis of Akt1, Akt2, Akt3, and GAPDH in MCF7 cells expressing shLuciferase, shBtn1a1 or shAkt1. (TIF) [file pone.0024432.s002.tif]
