# Supplementary material for: Akt1 Is Essential for Postnatal Mammary Gland Development, Function, and the Expression of Btn1a1
Source: PLoS One. 2011 Sep 7;6(9):e24432. doi: 10.1371/journal.pone.0024432 (PMC3168520; doi:10.1371/journal.pone.0024432)
Supplement: Table S1 — Differentially expressed gene list in Akt1−/− mammary glands. Listed are the statistically significant genes with a p<0.05 and a fold-change of <1.5 in adult Akt1−/− mammary glands compared to Akt1+/+ mammary glands. (DOC) [file pone.0024432.s003.doc]

**Table S1: Differentially expressed gene list in *Akt1-/-* mammary glands.**

| Column # | Probeset ID | gene_assignment | Gene Symbol | RefSeq | p-value(Genotype) | p-value(KO vs. WT) | Ratio(KO vs. WT) | Fold-Change(KO vs. WT) | Fold-Change(KO vs. WT) (Description) | F(Genotype) | F(Error) |
| --- | --- | --- | --- | --- | --- | --- | --- | --- | --- | --- | --- |
| 6697 | 10408175 | NM_013483 // Btn1a1 // butyrophilin, subfamily 1, member A1 // 13 A3.1|13 12.0 c | Btn1a1 | NM_013483 | 0.0327521 | 0.0327521 | 0.359983 | -2.77791 | KO down vs WT | 10.2705 | 1 |
| 6009 | 10402766 | NM_009652 // Akt1 // thymoma viral proto-oncogene 1 // 12 F1|12 F1-F2|12 57.0 cM | Akt1 | NM_009652 | 0.000996112 | 0.000996112 | 0.395553 | -2.52811 | KO down vs WT | 74.2883 | 1 |
| 26368 | 10587778 | --- |  | --- | 0.0495915 | 0.0495915 | 0.421416 | -2.37295 | KO down vs WT | 7.75326 | 1 |
| 25296 | 10578300 | NM_001040699 // Mtmr7 // myotubularin related protein 7 // 8 B1.2 // 54384 /// E | Mtmr7 | NM_001040699 | 0.0445772 | 0.0445772 | 0.45395 | -2.20288 | KO down vs WT | 8.35025 | 1 |
| 6219 | 10404376 | NM_177322 // Agtr1a // angiotensin II receptor, type 1a // 13 A3.2|13 16.0 cM // | Agtr1a | NM_177322 | 0.00582721 | 0.00582721 | 0.466034 | -2.14577 | KO down vs WT | 28.782 | 1 |
| 13794 | 10474102 | --- |  | --- | 0.0381957 | 0.0381957 | 0.475148 | -2.10461 | KO down vs WT | 9.27511 | 1 |
| 5456 | 10398075 | NM_009252 // Serpina3n // serine (or cysteine) peptidase inhibitor, clade A, mem | Serpina3n | NM_009252 | 0.0134219 | 0.0134219 | 0.496993 | -2.0121 | KO down vs WT | 17.852 | 1 |
| 28579 | 10606792 | NM_130888 // Nxf7 // nuclear RNA export factor 7 // X F1|X 53.2 cM // 170722 /// | Nxf7 | NM_130888 | 0.042242 | 0.042242 | 0.507576 | -1.97015 | KO down vs WT | 8.66415 | 1 |
| 5240 | 10395869 | BC100489 // 4921506M07Rik // RIKEN cDNA 4921506M07 gene // 12 C1 // 70846 /// EN | 4921506M07Rik | BC100489 | 0.0334652 | 0.0334652 | 0.527711 | -1.89498 | KO down vs WT | 10.1263 | 1 |
| 8962 | 10428534 | NM_032000 // Trps1 // trichorhinophalangeal syndrome I (human) // 15 C|15 30.1 c | Trps1 | NM_032000 | 0.0321771 | 0.0321771 | 0.546108 | -1.83114 | KO down vs WT | 10.3902 | 1 |
| 9750 | 10436598 | ENSMUST00000068704 // 2810055G20Rik // RIKEN cDNA 2810055G20 gene // 16 C3.1 // | 2810055G20Rik | ENSMUST00000068704 | 0.0244938 | 0.0244938 | 0.553171 | -1.80776 | KO down vs WT | 12.3765 | 1 |
| 24601 | 10571444 | NM_007514 // Slc7a2 // solute carrier family 7 (cationic amino acid transporter, | Slc7a2 | NM_007514 | 0.0396216 | 0.0396216 | 0.567622 | -1.76174 | KO down vs WT | 9.0491 | 1 |
| 25110 | 10576586 | NM_175434 // Slc35f3 // solute carrier family 35, member F3 // 8 E2 // 210027 // | Slc35f3 | NM_175434 | 0.0378495 | 0.0378495 | 0.568043 | -1.76043 | KO down vs WT | 9.33192 | 1 |
| 17786 | 10511560 | NM_194055 // Esrp1 // epithelial splicing regulatory protein 1 // 4 A1 // 207920 | Esrp1 | NM_194055 | 0.0421517 | 0.0421517 | 0.569083 | -1.75721 | KO down vs WT | 8.6768 | 1 |
| 15799 | 10493474 | NM_013605 // Muc1 // mucin 1, transmembrane // 3 F1|3 44.8 cM // 17829 /// ENSMU | Muc1 | NM_013605 | 0.0125494 | 0.0125494 | 0.570806 | -1.75191 | KO down vs WT | 18.5732 | 1 |
| 23562 | 10563955 | XM_001475615 // Gm3079 // predicted gene 3079 // 7 C|7 // 100040985 | Gm3079 | XM_001475615 | 0.0483878 | 0.0483878 | 0.577579 | -1.73137 | KO down vs WT | 7.88801 | 1 |
| 5155 | 10394990 | NM_026037 // Mboat2 // membrane bound O-acyltransferase domain containing 2 // 1 | Mboat2 | NM_026037 | 0.00249916 | 0.00249916 | 0.580707 | -1.72204 | KO down vs WT | 45.6822 | 1 |
| 9341 | 10432554 | --- |  | --- | 0.0153121 | 0.0153121 | 0.592283 | -1.68838 | KO down vs WT | 16.5072 | 1 |
| 23927 | 10566211 | NM_147070 // Olfr604 // olfactory receptor 604 // 7 E3 // 259073 /// ENSMUST0000 | Olfr604 | NM_147070 | 0.0428045 | 0.0428045 | 0.598094 | -1.67198 | KO down vs WT | 8.58618 | 1 |
| 763 | 10352104 | ENSMUST00000111149 // Gm16432 // predicted gene 16432 // 1 H4 // 545391 | Gm16432 | ENSMUST00000111149 | 0.00786674 | 0.00786674 | 0.605227 | -1.65227 | KO down vs WT | 24.3156 | 1 |
| 27232 | 10596279 | NM_001163026 // Dnajc13 // DnaJ (Hsp40) homolog, subfamily C, member 13 // 9 F1 | Dnajc13 | NM_001163026 | 0.040134 | 0.040134 | 0.606316 | -1.64931 | KO down vs WT | 8.97086 | 1 |
| 796 | 10352416 | NM_130890 // Capn8 // calpain 8 // 1 H4 // 170725 /// NM_001145806 // Capn8 // c | Capn8 | NM_130890 | 0.00474826 | 0.00474826 | 0.607733 | -1.64546 | KO down vs WT | 32.2385 | 1 |
| 21071 | 10542965 | NM_001130190 // Sgce // sarcoglycan, epsilon // 6 A1|6 1.0 cM // 20392 /// NM_00 | Sgce | NM_001130190 | 0.0347747 | 0.0347747 | 0.610987 | -1.6367 | KO down vs WT | 9.87329 | 1 |
| 27938 | 10601848 | NR_003641 // 6530401D17Rik // signal peptidase complex subunit 3 homolog pseudog | 6530401D17Rik | NR_003641 | 0.0364638 | 0.0364638 | 0.612024 | -1.63392 | KO down vs WT | 9.56737 | 1 |
| 8780 | 10427075 | NM_010664 // Krt18 // keratin 18 // 15 F3|15 58.86 cM // 16668 /// ENSMUST000000 | Krt18 | NM_010664 | 0.0476933 | 0.0476933 | 0.616941 | -1.6209 | KO down vs WT | 7.96809 | 1 |
| 27364 | 10597817 | NM_031161 // Cck // cholecystokinin // 9 F4|9 71.0 cM // 12424 /// ENSMUST000000 | Cck | NM_031161 | 0.0327722 | 0.0327722 | 0.617681 | -1.61896 | KO down vs WT | 10.2663 | 1 |
| 10933 | 10447383 | NM_008532 // Epcam // epithelial cell adhesion molecule // 17 E4 // 17075 /// EN | Epcam | NM_008532 | 0.0393121 | 0.0393121 | 0.624047 | -1.60244 | KO down vs WT | 9.0971 | 1 |
| 7364 | 10414179 | --- |  | --- | 0.00841739 | 0.00841739 | 0.624591 | -1.60105 | KO down vs WT | 23.3981 | 1 |
| 17660 | 10510230 | NM_001039209 // Gm13152 // predicted gene 13152 // 4 E1|4 // 195531 /// NM_00101 | Gm13152 | NM_001039209 | 0.03022 | 0.03022 | 0.630097 | -1.58706 | KO down vs WT | 10.8231 | 1 |
| 20259 | 10535807 | NM_010228 // Flt1 // FMS-like tyrosine kinase 1 // 5 G|5 82.0 cM // 14254 /// EN | Flt1 | NM_010228 | 0.0227991 | 0.0227991 | 0.632611 | -1.58075 | KO down vs WT | 12.9455 | 1 |
| 16657 | 10501020 | NM_009892 // Chi3l3 // chitinase 3-like 3 // 3 F2.2|3 50.5 cM // 12655 /// ENSMU | Chi3l3 | NM_009892 | 0.015893 | 0.015893 | 0.641635 | -1.55852 | KO down vs WT | 16.1429 | 1 |
| 16942 | 10503502 | NM_015767 // Ttpa // tocopherol (alpha) transfer protein // 4 A3|4 22.7 cM // 50 | Ttpa | NM_015767 | 0.0316982 | 0.0316982 | 0.642272 | -1.55697 | KO down vs WT | 10.4924 | 1 |
| 6549 | 10407122 | --- |  | --- | 0.0251376 | 0.0251376 | 0.644814 | -1.55083 | KO down vs WT | 12.1756 | 1 |
| 7789 | 10417235 | EF651820 // Gm3264 // predicted gene 3264 // 14 A1|14 // 100041306 /// CT010181 | Gm3264 | EF651820 | 0.0121669 | 0.0121669 | 0.64629 | -1.54729 | KO down vs WT | 18.9136 | 1 |
| 7802 | 10417315 | EF651820 // Gm3264 // predicted gene 3264 // 14 A1|14 // 100041306 /// EF651808 | Gm3264 | EF651820 | 0.0121669 | 0.0121669 | 0.64629 | -1.54729 | KO down vs WT | 18.9136 | 1 |
| 12193 | 10459353 | --- |  | --- | 0.0333374 | 0.0333374 | 0.64882 | -1.54126 | KO down vs WT | 10.1518 | 1 |
| 7733 | 10416800 | NM_201529 // Lmo7 // LIM domain only 7 // 14 E2.3 // 380928 /// ENSMUST000001003 | Lmo7 | NM_201529 | 0.0327205 | 0.0327205 | 0.65064 | -1.53695 | KO down vs WT | 10.2769 | 1 |
| 11981 | 10457357 | NM_001081287 // Mpp7 // membrane protein, palmitoylated 7 (MAGUK p55 subfamily m | Mpp7 | NM_001081287 | 0.0346447 | 0.0346447 | 0.651556 | -1.53479 | KO down vs WT | 9.89776 | 1 |
| 17024 | 10504234 | NM_001081413 // Unc13b // unc-13 homolog B (C. elegans) // --- // 22249 /// ENSM | Unc13b | NM_001081413 | 0.0490714 | 0.0490714 | 0.652877 | -1.53168 | KO down vs WT | 7.81088 | 1 |
| 21875 | 10549917 | NM_198961 // Vmn2r43 // vomeronasal 2, receptor 43 // 7 A1 // 381838 /// NM_1989 | Vmn2r43 | NM_198961 | 0.00314382 | 0.00314382 | 0.653 | -1.53139 | KO down vs WT | 40.3729 | 1 |
| 13529 | 10472212 | NM_026361 // Pkp4 // plakophilin 4 // 2 C3 // 227937 /// NM_175464 // Pkp4 // pl | Pkp4 | NM_026361 | 0.0312515 | 0.0312515 | 0.657268 | -1.52145 | KO down vs WT | 10.5898 | 1 |
| 1351 | 10358259 | NM_021605 // Nek7 // NIMA (never in mitosis gene a)-related expressed kinase 7 / | Nek7 | NM_021605 | 0.044263 | 0.044263 | 0.659096 | -1.51723 | KO down vs WT | 8.39104 | 1 |
| 2575 | 10369102 | ENSMUST00000095691 // Gm9766 // predicted gene 9766 // 10|10 // 100038725 | Gm9766 | ENSMUST00000095691 | 0.015761 | 0.015761 | 0.660497 | -1.51401 | KO down vs WT | 16.2239 | 1 |
| 48 | 10344837 | NM_029525 // Prex2 // phosphatidylinositol-3,4,5-trisphosphate-dependent Rac exc | Prex2 | NM_029525 | 0.010302 | 0.010302 | 0.660959 | -1.51295 | KO down vs WT | 20.8366 | 1 |
| 26200 | 10585976 | NM_173018 // Myo9a // myosin IXa // 9 B // 270163 /// ENSMUST00000085572 // Myo9 | Myo9a | NM_173018 | 0.0120881 | 0.0120881 | 0.663742 | -1.50661 | KO down vs WT | 18.9857 | 1 |
| 8446 | 10423825 | NM_008056 // Fzd6 // frizzled homolog 6 (Drosophila) // 15 B3.1|15 13.1 cM // 14 | Fzd6 | NM_008056 | 0.0337547 | 0.0337547 | 0.664301 | -1.50534 | KO down vs WT | 10.0691 | 1 |
| 26807 | 10592266 | NM_001145960 // Slc37a2 // solute carrier family 37 (glycerol-3-phosphate transp | Slc37a2 | NM_001145960 | 0.0232237 | 0.0232237 | 1.50147 | 1.50147 | KO up vs WT | 12.7971 | 1 |
| 20335 | 10536401 | NM_008751 // Nxph1 // neurexophilin 1 // 6 A1 // 18231 /// ENSMUST00000060369 // | Nxph1 | NM_008751 | 0.0224396 | 0.0224396 | 1.50169 | 1.50169 | KO up vs WT | 13.0744 | 1 |
| 5477 | 10398286 | --- |  | --- | 0.0474375 | 0.0474375 | 1.50217 | 1.50217 | KO up vs WT | 7.99802 | 1 |
| 22921 | 10558769 | NM_026820 // Ifitm1 // interferon induced transmembrane protein 1 // 7 F5 // 687 | Ifitm1 | NM_026820 | 0.00237412 | 0.00237412 | 1.50315 | 1.50315 | KO up vs WT | 46.9555 | 1 |
| 24463 | 10570434 | NM_026820 // Ifitm1 // interferon induced transmembrane protein 1 // 7 F5 // 687 | Ifitm1 | NM_026820 | 0.00833573 | 0.00833573 | 1.5132 | 1.5132 | KO up vs WT | 23.5284 | 1 |
| 11297 | 10450731 | XR_034252 // H2-t9 // MHC class Ib T9 // 17 B1 // 630294 /// XR_034810 // H2-t9 | H2-t9 | XR_034252 | 0.0137669 | 0.0137669 | 1.51382 | 1.51382 | KO up vs WT | 17.586 | 1 |
| 6665 | 10408083 | NM_145073 // Hist1h3g // histone cluster 1, H3g // 13 A2-A3 // 97908 /// NM_0135 | Hist1h3g | NM_145073 | 0.0224271 | 0.0224271 | 1.51401 | 1.51401 | KO up vs WT | 13.079 | 1 |
| 6709 | 10408239 | NM_013548 // Hist1h3f // histone cluster 1, H3f // 13 A2-A3|13 12.0 cM // 260423 | Hist1h3f | NM_013548 | 0.0248647 | 0.0248647 | 1.52173 | 1.52173 | KO up vs WT | 12.2598 | 1 |
| 26288 | 10586907 | NM_008613 // Mns1 // meiosis-specific nuclear structural protein 1 // 9 D // 174 | Mns1 | NM_008613 | 0.0401436 | 0.0401436 | 1.52312 | 1.52312 | KO up vs WT | 8.96939 | 1 |
| 875 | 10353192 | NM_010164 // Eya1 // eyes absent 1 homolog (Drosophila) // 1 A3|1 10.4 cM // 140 | Eya1 | NM_010164 | 0.0144067 | 0.0144067 | 1.52386 | 1.52386 | KO up vs WT | 17.1182 | 1 |
| 6177 | 10404049 | NM_145073 // Hist1h3g // histone cluster 1, H3g // 13 A2-A3 // 97908 /// NM_1782 | Hist1h3g | NM_145073 | 0.0277513 | 0.0277513 | 1.5263 | 1.5263 | KO up vs WT | 11.4334 | 1 |
| 6135 | 10403941 | NM_145073 // Hist1h3g // histone cluster 1, H3g // 13 A2-A3 // 97908 /// NM_0135 | Hist1h3g | NM_145073 | 0.0231771 | 0.0231771 | 1.52726 | 1.52726 | KO up vs WT | 12.8131 | 1 |
| 25661 | 10581650 | NM_011998 // Chst4 // carbohydrate (chondroitin 6/keratan) sulfotransferase 4 // | Chst4 | NM_011998 | 0.0389725 | 0.0389725 | 1.53103 | 1.53103 | KO up vs WT | 9.15044 | 1 |
| 24367 | 10569341 | NR_001592 // H19 // H19 fetal liver mRNA // 7 F5|7 69.03 cM // 14955 | H19 | NR_001592 | 0.0239571 | 0.0239571 | 1.53458 | 1.53458 | KO up vs WT | 12.5501 | 1 |
| 7928 | 10418729 | NM_009937 // Colq // collagen-like tail subunit (single strand of homotrimer) of | Colq | NM_009937 | 0.047758 | 0.047758 | 1.53634 | 1.53634 | KO up vs WT | 7.96055 | 1 |
| 6184 | 10404065 | NM_013548 // Hist1h3f // histone cluster 1, H3f // 13 A2-A3|13 12.0 cM // 260423 | Hist1h3f | NM_013548 | 0.0299367 | 0.0299367 | 1.54297 | 1.54297 | KO up vs WT | 10.8892 | 1 |
| 17278 | 10506301 | NM_001122899 // Lepr // leptin receptor // 4 C6|4 46.7 cM // 16847 /// NM_146146 | Lepr | NM_001122899 | 0.0152523 | 0.0152523 | 1.54505 | 1.54505 | KO up vs WT | 16.5459 | 1 |
| 7477 | 10414811 | ENSMUST00000103645 // Gm13969 // predicted gene 13969 // 14 C2 // 667481 | Gm13969 | ENSMUST00000103645 | 0.0225607 | 0.0225607 | 1.54514 | 1.54514 | KO up vs WT | 13.0306 | 1 |
| 7505 | 10414937 | ENSMUST00000103645 // Gm13969 // predicted gene 13969 // 14 C2 // 667481 | Gm13969 | ENSMUST00000103645 | 0.0225607 | 0.0225607 | 1.54514 | 1.54514 | KO up vs WT | 13.0306 | 1 |
| 23789 | 10565218 | NM_010551 // Il16 // interleukin 16 // 7 D2-D3|7 41.2 cM // 16170 /// ENSMUST000 | Il16 | NM_010551 | 0.0324918 | 0.0324918 | 1.54667 | 1.54667 | KO up vs WT | 10.3243 | 1 |
| 4092 | 10384223 | NM_008343 // Igfbp3 // insulin-like growth factor binding protein 3 // 11 A1|11 | Igfbp3 | NM_008343 | 0.0292111 | 0.0292111 | 1.56157 | 1.56157 | KO up vs WT | 11.0631 | 1 |
| 1604 | 10360306 | NM_029084 // Slamf8 // SLAM family member 8 // 1 H2 // 74748 /// ENSMUST00000065 | Slamf8 | NM_029084 | 0.046877 | 0.046877 | 1.56264 | 1.56264 | KO up vs WT | 8.06448 | 1 |
| 3790 | 10381154 | NM_009923 // Cnp // 2',3'-cyclic nucleotide 3' phosphodiesterase // 11 D|11 60.0 | Cnp | NM_009923 | 0.0349419 | 0.0349419 | 1.56442 | 1.56442 | KO up vs WT | 9.84202 | 1 |
| 1468 | 10358845 | ENSMUST00000097535 // Gm6648 // predicted gene 6648 // 1 G3 // 626058 | Gm6648 | ENSMUST00000097535 | 0.0141302 | 0.0141302 | 1.57128 | 1.57128 | KO up vs WT | 17.3165 | 1 |
| 25065 | 10576140 | NM_026014 // Cdt1 // chromatin licensing and DNA replication factor 1 // 8 E1 // | Cdt1 | NM_026014 | 0.0366314 | 0.0366314 | 1.57144 | 1.57144 | KO up vs WT | 9.53818 | 1 |
| 21835 | 10549647 | NM_010746 // Ncr1 // natural cytotoxicity triggering receptor 1 // 7 A1 // 17086 | Ncr1 | NM_010746 | 0.0402392 | 0.0402392 | 1.57174 | 1.57174 | KO up vs WT | 8.95498 | 1 |
| 8527 | 10424607 | NM_008975 // Ptp4a3 // protein tyrosine phosphatase 4a3 // 15 E1 // 19245 /// EN | Ptp4a3 | NM_008975 | 0.0489837 | 0.0489837 | 1.57739 | 1.57739 | KO up vs WT | 7.82068 | 1 |
| 8274 | 10421911 | NM_178685 // Pcdh20 // protocadherin 20 // 14 E1 // 219257 /// ENSMUST0000006162 | Pcdh20 | NM_178685 | 0.0232929 | 0.0232929 | 1.58474 | 1.58474 | KO up vs WT | 12.7732 | 1 |
| 13133 | 10468294 | NM_133746 // Calhm2 // calcium homeostasis modulator 2 // 19 C3 // 72691 /// ENS | Calhm2 | NM_133746 | 0.0204656 | 0.0204656 | 1.58748 | 1.58748 | KO up vs WT | 13.8421 | 1 |
| 901 | 10353420 | NM_008563 // Mcm3 // minichromosome maintenance deficient 3 (S. cerevisiae) // 1 | Mcm3 | NM_008563 | 0.0403114 | 0.0403114 | 1.59067 | 1.59067 | KO up vs WT | 8.94411 | 1 |
| 7829 | 10417568 | NM_146050 // Oit1 // oncoprotein induced transcript 1 // 14 A1 // 18300 /// ENSM | Oit1 | NM_146050 | 0.0116472 | 0.0116472 | 1.60318 | 1.60318 | KO up vs WT | 19.4027 | 1 |
| 15922 | 10494405 | NM_178215 // Hist2h3b // histone cluster 2, H3b // 3 F2.1 // 319154 /// NM_01946 | Hist2h3b | NM_178215 | 0.0277546 | 0.0277546 | 1.60632 | 1.60632 | KO up vs WT | 11.4325 | 1 |
| 15756 | 10492964 | NM_009690 // Cd5l // CD5 antigen-like // 3 F1 // 11801 /// ENSMUST00000015998 // | Cd5l | NM_009690 | 0.0371136 | 0.0371136 | 1.60667 | 1.60667 | KO up vs WT | 9.45531 | 1 |
| 28593 | 10606910 | NM_001082412 // Mcart6 // mitochondrial carrier triple repeat 6 // X F1 // 67062 | Mcart6 | NM_001082412 | 0.0276149 | 0.0276149 | 1.61101 | 1.61101 | KO up vs WT | 11.4695 | 1 |
| 4172 | 10384974 | NM_001134458 // Il9r // interleukin 9 receptor // 11 A4 // 16199 /// NM_008374 / | Il9r | NM_001134458 | 0.0376198 | 0.0376198 | 1.65212 | 1.65212 | KO up vs WT | 9.37005 | 1 |
| 2793 | 10371506 | NM_138673 // Stab2 // stabilin 2 // 10 C1 // 192188 /// ENSMUST00000035288 // St | Stab2 | NM_138673 | 0.0420158 | 0.0420158 | 1.67051 | 1.67051 | KO up vs WT | 8.69595 | 1 |
| 7641 | 10416057 | NM_013492 // Clu // clusterin // 14 D1|14 28.0 cM // 12759 /// ENSMUST0000002261 | Clu | NM_013492 | 0.0398257 | 0.0398257 | 1.69712 | 1.69712 | KO up vs WT | 9.01776 | 1 |
| 18921 | 10522288 | NM_001033415 // Shisa3 // shisa homolog 3 (Xenopus laevis) // 5 C3.1 // 330096 / | Shisa3 | NM_001033415 | 0.0095597 | 0.0095597 | 1.70387 | 1.70387 | KO up vs WT | 21.7546 | 1 |
| 24366 | 10569335 | NR_001592 // H19 // H19 fetal liver mRNA // 7 F5|7 69.03 cM // 14955 | H19 | NR_001592 | 0.026165 | 0.026165 | 1.7094 | 1.7094 | KO up vs WT | 11.8706 | 1 |
| 6021 | 10402991 | ENSMUST00000103443 // Ighvq52.3.8 // immunoglobulin heavy chain variable region | Ighvq52.3.8 | ENSMUST00000103443 | 0.0164266 | 0.0164266 | 1.76507 | 1.76507 | KO up vs WT | 15.8256 | 1 |
| 12294 | 10460237 | NM_019449 // Unc93b1 // unc-93 homolog B1 (C. elegans) // 19 A // 54445 /// NM_0 | Unc93b1 | NM_019449 | 0.0367369 | 0.0367369 | 1.78272 | 1.78272 | KO up vs WT | 9.51991 | 1 |
| 3457 | 10377826 | NM_001029929 // Zmynd15 // zinc finger, MYND-type containing 15 // 11 B3 // 5744 | Zmynd15 | NM_001029929 | 0.0289112 | 0.0289112 | 1.80051 | 1.80051 | KO up vs WT | 11.1369 | 1 |
| 28116 | 10603440 | NM_009515 // Was // Wiskott-Aldrich syndrome homolog (human) // X A1.1|X 2.0 cM | Was | NM_009515 | 0.0315968 | 0.0315968 | 1.80162 | 1.80162 | KO up vs WT | 10.5143 | 1 |
| 5452 | 10398039 | NM_001033335 // Serpina3f // serine (or cysteine) peptidase inhibitor, clade A, | Serpina3f | NM_001033335 | 0.0312816 | 0.0312816 | 1.81223 | 1.81223 | KO up vs WT | 10.5832 | 1 |
| 24672 | 10572050 | NM_175188 // March1 // membrane-associated ring finger (C3HC4) 1 // 8 B3.1-B3.2 | March1 | NM_175188 | 0.036041 | 0.036041 | 1.81713 | 1.81713 | KO up vs WT | 9.64191 | 1 |
| 7511 | 10414955 | ENSMUST00000103654 // OTTMUSG00000015050 // predicted gene, OTTMUSG00000015050 / | OTTMUSG00000015050 | ENSMUST00000103654 | 0.0488907 | 0.0488907 | 1.82106 | 1.82106 | KO up vs WT | 7.8311 | 1 |
| 23403 | 10562709 | NM_001111058 // Cd33 // CD33 antigen // 7 B4|7 23.0 cM // 12489 /// NM_021293 // | Cd33 | NM_001111058 | 0.0467356 | 0.0467356 | 1.8242 | 1.8242 | KO up vs WT | 8.08144 | 1 |
| 25704 | 10582162 | NM_028071 // Cotl1 // coactosin-like 1 (Dictyostelium) // 8 E1 // 72042 /// ENSM | Cotl1 | NM_028071 | 0.0426575 | 0.0426575 | 1.86065 | 1.86065 | KO up vs WT | 8.60641 | 1 |
| 25414 | 10579347 | NM_023065 // Ifi30 // interferon gamma inducible protein 30 // 8 B3.3 // 65972 / | Ifi30 | NM_023065 | 0.0237065 | 0.0237065 | 1.87206 | 1.87206 | KO up vs WT | 12.6332 | 1 |
| 429 | 10348902 | NM_199366 // Gal3st2 // galactose-3-O-sulfotransferase 2 // 1 D // 381334 /// NM | Gal3st2 | NM_199366 | 0.0497554 | 0.0497554 | 1.88664 | 1.88664 | KO up vs WT | 7.73529 | 1 |
| 10689 | 10445119 | NM_013819 // H2-M3 // histocompatibility 2, M region locus 3 // 17 B1|17 20.38 c | H2-M3 | NM_013819 | 0.04324 | 0.04324 | 1.91615 | 1.91615 | KO up vs WT | 8.52686 | 1 |
| 10598 | 10444229 | NM_010386 // H2-DMa // histocompatibility 2, class II, locus DMa // 17 B1|17 18. | H2-DMa | NM_010386 | 0.0452878 | 0.0452878 | 1.97152 | 1.97152 | KO up vs WT | 8.25962 | 1 |
| 10605 | 10444298 | NM_010382 // H2-Eb1 // histocompatibility 2, class II antigen E beta // 17 B1|17 | H2-Eb1 | NM_010382 | 0.0378854 | 0.0378854 | 2.01856 | 2.01856 | KO up vs WT | 9.326 | 1 |
| 1267 | 10357261 | NM_010766 // Marco // macrophage receptor with collagenous structure // 1 E4-F / | Marco | NM_010766 | 0.0442079 | 0.0442079 | 2.03001 | 2.03001 | KO up vs WT | 8.39822 | 1 |
| 14096 | 10476945 | NM_009977 // Cst7 // cystatin F (leukocystatin) // 2 G1-G3 // 13011 /// ENSMUST0 | Cst7 | NM_009977 | 0.0466174 | 0.0466174 | 2.03849 | 2.03849 | KO up vs WT | 8.09567 | 1 |
| 2759 | 10371217 | NM_010102 // S1pr4 // sphingosine-1-phosphate receptor 4 // 10 C1 // 13611 /// E | S1pr4 | NM_010102 | 0.0337815 | 0.0337815 | 2.04532 | 2.04532 | KO up vs WT | 10.0638 | 1 |
| 5041 | 10394054 | NM_009854 // Cd7 // CD7 antigen // 11 E2|11 74.0 cM // 12516 /// ENSMUST00000026 | Cd7 | NM_009854 | 0.0431219 | 0.0431219 | 2.08498 | 2.08498 | KO up vs WT | 8.54287 | 1 |
| 11236 | 10450154 | NM_010378 // H2-Aa // histocompatibility 2, class II antigen A, alpha // 17 B1|1 | H2-Aa | NM_010378 | 0.0356223 | 0.0356223 | 2.0863 | 2.0863 | KO up vs WT | 9.71705 | 1 |
| 22808 | 10557571 | BC038694 // AI467606 // expressed sequence AI467606 // 7 F3 // 101602 /// NM_178 | AI467606 | BC038694 | 0.0466378 | 0.0466378 | 2.18669 | 2.18669 | KO up vs WT | 8.09321 | 1 |
| 1771 | 10361906 | NM_178258 // Il22ra2 // interleukin 22 receptor, alpha 2 // 10 A3 // 237310 /// | Il22ra2 | NM_178258 | 0.0131625 | 0.0131625 | 2.21057 | 2.21057 | KO up vs WT | 18.0589 | 1 |
| 18860 | 10521667 | NM_009763 // Bst1 // bone marrow stromal cell antigen 1 // 5 B3|5 25.0 cM // 121 | Bst1 | NM_009763 | 0.0422926 | 0.0422926 | 2.24428 | 2.24428 | KO up vs WT | 8.65706 | 1 |
| 23884 | 10565924 | NM_177073 // Relt // RELT tumor necrosis factor receptor // 7 E3 // 320100 /// E | Relt | NM_177073 | 0.033465 | 0.033465 | 2.24997 | 2.24997 | KO up vs WT | 10.1263 | 1 |
| 10604 | 10444291 | NM_207105 // H2-Ab1 // histocompatibility 2, class II antigen A, beta 1 // 17 B1 | H2-Ab1 | NM_207105 | 0.0248715 | 0.0248715 | 2.25924 | 2.25924 | KO up vs WT | 12.2577 | 1 |
| 6117 | 10403821 | ENSMUST00000103558 // Tcrg-V3 // T-cell receptor gamma, variable 3 // --- // 216 | Tcrg-V3 | ENSMUST00000103558 | 0.0409879 | 0.0409879 | 2.30081 | 2.30081 | KO up vs WT | 8.84375 | 1 |
| 10876 | 10446763 | NM_029999 // Lbh // limb-bud and heart // --- // 77889 /// BC052470 // Lbh // li | Lbh | NM_029999 | 0.0497461 | 0.0497461 | 2.40299 | 2.40299 | KO up vs WT | 7.73631 | 1 |
| 6633 | 10407940 | ENSMUST00000103563 // Naip3 // NLR family, apoptosis inhibitory protein 3 // 13 | Naip3 | ENSMUST00000103563 | 0.0492682 | 0.0492682 | 2.617 | 2.617 | KO up vs WT | 7.78896 | 1 |
| 2895 | 10372652 | NM_013590 // Lyz1 // lysozyme 1 // 10 D2 // 17110 /// ENSMUST00000092162 // Lyz1 | Lyz1 | NM_013590 | 0.0480298 | 0.0480298 | 2.99895 | 2.99895 | KO up vs WT | 7.92906 | 1 |
| 10597 | 10444223 | NM_008206 // H2-Oa // histocompatibility 2, O region alpha locus // 17 B1|17 18. | H2-Oa | NM_008206 | 0.0477931 | 0.0477931 | 3.1921 | 3.1921 | KO up vs WT | 7.95647 | 1 |
| 12014 | 10457669 | NM_007882 // Dsc3 // desmocollin 3 // 18 A2|18 7.0 cM // 13507 /// ENSMUST000001 | Dsc3 | NM_007882 | 0.0488887 | 0.0488887 | 3.31179 | 3.31179 | KO up vs WT | 7.83133 | 1 |
| 26126 | 10585276 | NM_011136 // Pou2af1 // POU domain, class 2, associating factor 1 // 9 A5.3 // 1 | Pou2af1 | NM_011136 | 0.0466374 | 0.0466374 | 3.7606 | 3.7606 | KO up vs WT | 8.09325 | 1 |
| 498 | 10349593 | NM_026976 // Faim3 // Fas apoptotic inhibitory molecule 3 // 1 E4 // 69169 /// E | Faim3 | NM_026976 | 0.0407498 | 0.0407498 | 4.54835 | 4.54835 | KO up vs WT | 8.87878 | 1 |
| 9947 | 10438405 | M94350 // Igl-V1 // immunoglobulin lambda chain, variable 1 // 16 A3|16 13.0 cM | Igl-V1 | M94350 | 0.0451743 | 0.0451743 | 5.00615 | 5.00615 | KO up vs WT | 8.27395 | 1 |
| 20609 | 10538871 | --- |  | --- | 0.0443432 | 0.0443432 | 5.25344 | 5.25344 | KO up vs WT | 8.38058 | 1 |
